# Measuring the microbiome of chronic wounds with use of a topical antimicrobial dressing – A feasibility study

**DOI:** 10.1371/journal.pone.0187728

**Published:** 2017-11-20

**Authors:** Lindsay Kalan, Mi Zhou, Michele Labbie, Benjamin Willing

**Affiliations:** 1 Exciton Technologies Inc, Edmonton, Alberta, Canada; 2 Department of Agricultural, Food and Nutritional Science, University of Alberta, Edmonton, Alberta, Canada; 3 Westview Health Center, Stony Plain, Alberta, Canada; Rush University, UNITED STATES

## Abstract

**Background:**

Polymicrobial communities colonize all wounds, and biofilms are hypothesized to be a key link to the chronic state and stalled healing. Molecular methods offer greater insight when studying microbial ecology in chronic wounds, as only a small fraction of wound bacteria are cultured by currently available methods and studies have shown little agreement between culture and molecular based approaches. Some interventions, like dressings with oxidized silver, are reported to help the stalled wounds move to a normal healing trajectory but the underlying mechanisms are difficult to measure. One hypothesis is that the use of topical antimicrobial dressings targets the wound microbiome and reduces bioburden.

**Objectives:**

Our objective was to determine if culture-independent molecular methods could be used to identify the microbial composition in chronic wounds, and measure the microbiome over time when a topical antimicrobial dressing is used to reduce bioburden.

**Methods:**

Patients with chronic wounds defined as >6 weeks in duration and not taking systemic antibiotics were recruited to participate. A wound contact layer containing silver oxynitrate was applied immediately after routine sharp debridement material was collected and swabs of the wound bed taken. Next-generation sequencing of the bacterial 16S rRNA gene in each specimen was used to measure the microbiome.

**Results:**

Distinct bacterial communities were observed between swab and debridement samples, highlighting spatial differences and the importance of sampling consistency. The microbial communities appeared to be similar between different diabetes statuses, but different among the three wound categories included.

**Conclusions:**

Culture-independent methods can be applied to measure the microbiome of chronic wounds even when a topical antimicrobial dressing is applied to the wound.

## Introduction

During normal wound healing, the process that leads to tissue regeneration results from a series of tightly regulated sequential events[1,2]. In the case of chronic, non-healing wounds, this process is disrupted, leading to a prolonged inflammatory response and stalled healing. It is hypothesized that microbial colonization and the formation of a biofilm within the wound bed is positively associated with the transition from the acute to chronic state even without classical signs of infection[3]. When a biofilm forms, the penetration of some antimicrobials is reduced, while some individual members shift their metabolism to a more dormant state that can render other antibiotics ineffective. Treatment strategies are further complicated by co-morbidities that affect circulation such as diabetes, poor perfusion, and malnutrition, increasing the risk of infection and reducing the success of orally administered antibiotics[3].

The extent of microbial influences on tissue regeneration remains to be resolved. The skin itself is a diverse ecosystem harboring microbial populations that function to regulate host immune responses. Dysbiosis of the skin microbiota is associated with infection and disease and until recently the diversity of microbial communities within a chronic wound environment was poorly understood[4–10]. Advanced wound care treatments aim to restore wound balance and re-activate stalled healing pathways with topical antimicrobials targeting a reduction in bioburden and disruption of biofilm[3].

Silver has been used since antiquity as an antimicrobial agent for water purification[11]. Records of silver use for the treatment of wounds is recorded as early as the 19^th^ century, when application of silver sutures, foil, and silver nitrate were used to treat ulcers and surgical wounds[12–15]. The use of silver in medicine declined with the introduction of antibiotics, but as the threat of resistance continues to rise[16,17] silver is again becoming an important option, particularly in the treatment of chronic wounds. Silver (Ag_(*s*)_) in its elemental form is not antimicrobial, but must be oxidized to the silver ion Ag^+^ to exert its antimicrobial effects. AgNO_3_ is a ready source of Ag^+^ ions and is primarily used to treat gonococcal ophthalmia in neonates[18–20]. Like other transition metals, Ag^+^ salts are often insoluble precipitates (most notably AgCl_(*s*)_ and Ag_2_SO_4(*s*)_) or silver oxide (Ag_2_O_*(s)*_). Recently, the silver oxynitrate (Ag_7_NO_11*(s)*_) compound with Ag^2+^ and Ag^3+^ oxidation states has been described as having greater efficacy against both planktonic and biofilm growing bacteria than Ag^+^ compounds[21–23].

While many antimicrobial wound dressings claim activity against common skin pathogens such as *Staphylococcus aureus* or *Pseudomonas aeruginosa*, these claims are typically substantiated by *in vitro* methods that measure susceptibility by zone of inhibition or log reduction kill assays[24–27]. *In vivo* animal wound models may be more clinically relevant for assessing antimicrobial efficacy of wound dressings, however the cost associated with animal models limits the throughput, and the polymicrobial nature of the wound ecosystem is not truly represented. Indeed, with the increasing recognition of the human microbiome’s role in health and disease, many groups have begun to profile the wound microbiota in an effort to dissect the interactions between those microbes and the host response[4,5,7,9,28–32]. It is clear from these studies that a chronic wound is host to diverse communities of microbes that differ between individuals, however there have been few reports examining if the microbial composition can be measured over subsequent time points during the course of antimicrobial intervention.

This feasibility study set out with the aim to observe the microbiome of non-healing wounds longitudinally while a topical antimicrobial dressing was in use. Samples were analyzed by sequencing the bacterial 16S ribosomal RNA (rRNA) gene for microbial composition and diversity at baseline, prior to dressing application, within 24 hrs, and then weekly for up to four weeks or wound closure. In addition, both sharp debrided material and swabs of a cleansed wound bed were analyzed to determine if the sampling methodology influences the reported composition and relative abundance of individual genera. Here we report our findings as a feasibility study for future evaluation of the chronic wound microbiome by 16S rRNA sequencing.

## Materials and methods

### Sample collection

Debridement and swab samples were collected from wounds from 13 patients (ID: 1–13) at the Westview Health Center Wound Care Clinic (Stony Plain, AB, Canada) to determine the feasibility of measuring changes to the microbiome of chronic wounds during treatment with a topical antimicrobial agent. 15 patients were initially recruited but two subjects were removed. One patient was removed due to an MRSA+ culture and required systemic antibiotics. The second patient felt the dressing was uncomfortable and was discontinued after the first visit. Any data collected from those patients was not used. The study protocol was approved by the University of Alberta Health Research Ethics Board 3 (HREB 3) under the protocol number Pro00042930 (S2 File). The study is registered with ClinicalTrials.gov with ID NCT02662101. The study was not registered with ClinicalTrials.gov prior to patient enrollment because it was considered an observational study by the HREB. The intervention is a Health Canada and FDA approved medical device already in use in the study clinic. The intent of this feasibility study was to analyse samples collected as part of the standard of care in the wound clinic when the specified wound dressing are in use and determine the microbiome over time. The authors confirm that there are no ongoing or related trials but future studies will be registered prior to enrollment.

Subjects were enrolled and completed assessments between January 1, 2014 and August 31, 2014 at the Westview Health Center, Stony Plain, AB. All subjects were required to provide written informed consent to have samples taken and health data collected. Subjects, along with descriptive wound types and detailed health status, are listed in Table 1. Inclusion criteria included: 18 years of age or older; non-healing wounds defined as >6 weeks in duration; free of systemic antibiotics in the previous 2 weeks; and at least weekly visits for assessment required. At the first visit (V1), sharp debrided material and swabs taken by the Levine technique[33,34] post debridement and cleansing with sterile saline were collected. Post-sample collection, wound dressings containing silver oxynitrate were applied as a contact layer (exsalt SD7 or exsalt T7 Wound Dressings, Exciton Technologies, AB, Canada) with variable secondary dressings and securement depending on characteristics of individual wounds. Each subject was asked to return within 24–48 hrs to have an additional swab sample taken (V2). The subjects were then required to visit the clinic once a week for up to a maximum of four weeks to have debridement/swab samples collected as at baseline (V3-V6). Dressing changes occurred as needed for each individual case. All of the collected samples were put on ice immediately and transferred to -20°C for storage until further processing. The primary evaluation parameter was to determine if the wound microbiome can be detected with molecular (sequencing) based methods over subsequent visits. The feasibility criteria will be based on successful PCR amplification of the bacterial 16S rRNA gene and reconstruction of the microbiome in wound specimens by DNA sequencing of each amplicon.

**Table 1:** Patient demographics and wound characteristics at baseline (T = 0) and the end of study (T = 4).

Patient	Age/ Gender	Wound Location and Type	Wound Duration	Wound Area T = 0 (cm^2^)	Wound Area T = 4 wks (cm^2^)	% Closure	Underlying Disease
**1**	78/F	Lower leg VLU*	2 mo	3.20	0.40	87.5	Diabetes, Hypertension
**2**	72/F	Lower anterior resection complication	6 mo	2.80	1.16	58.6	Hypertension
**3**	69/M	Lateral foot DFU	3 mo	0.80	0.36	55.0	Diabetes
**4**	60/M	Skin tear of right flank	6 wks	0.36	0.00	100.0	None Recorded
**5**	66/M	Medial foot DFU	Several yrs	0.48	0.91	-89.6	Diabetes
**6**	47/M	Anterior toe joints	> 6 wks	0.20	0.00	100.0	CAD
**7**	70/M	Lateral foot DFU	> 6 wks	0.24	0.00	100.0	Diabetes
**8**	43/M	Medial foot VLU	5–8 yrs	4.56	0.99	78.3	Liver disease
**9**	67/M	Medial foot DFU	5 mo	0.60	0.24	60.0	Diabetes, Hypertension
**10**	66/F	Abdomen hernia repair complication	5 mo	64.80	0.00	100.0	Diabetes, Hypertension
**11**	71/F	Lower leg trauma wound	6 wks	37.50	8.50	77.3	None recorded
**12**	74/F	Surgical site infection to abdomen	6 wks	1.52	0.35	77.0	None Recorded
**13**	49/F	Lower leg VLU	>6wks	47.60	59.40	-24.7	None Recorded

## Protocol deviations

The criteria for subjects not to have been administered systemic antibiotics was reduced from 3 months to two weeks due to difficulty in identifying subjects that meet this criterion.

### Wound scoring

Wound scores were calculated at the end of the study by using all of the recorded metadata and photographs of the wounds from each visit that was collected for each subject at each time point and used the validated Bates-Jenson Wound Assessment Tool (BWAT)[35,36], a standardized tool used to generate a wound score indicating the wound progression. This score was recorded for each patient at each visit.

### DNA extraction, PCR amplification and 454 pyrosequencing

Total DNA was isolated from wound specimens following the methods described by Grice et al 2009. Briefly, specimens were subjected to DNA extraction using PureLink^®^ Genomic DNA kit (Thermo Scientific) following the protocol for Gram-positive bacteria pure culture. The DNA was diluted to 25 ng/μl template to amplify partial bacterial 16S rRNA gene fragments (V1-V3 region) with 27F (5’-TGCTGCCTCCCGTAGGAGT-3’) / 519R (5’-AGAGTTTGATCCTGGCTCAG-3’)[37]. The primary PCR reaction system (20 μl) included 1 μl of template, 0.4 μl of 10 mM deoxynucleoside triphosphate, 1 U of Thermo Scientific™ Phusion™ Hot Start II High-Fidelity DNA Polymerase (Fisher Scientific), 1× PCR buffer, 0.4 μl of 10 mM dNTP, 0.4 μl of 20 pmol of each primer (unlabeled), and nuclease-free water. The reaction program was: an initial denaturation for 1 min at 98°C; 20 cycles at 98°C for 10 s, 59°C for 30 s, and 72°C for 30 s; and a final elongation for 7 min at 72°C. The amplicons were then diluted 20 times to serve as the template for the secondary PCR. The system and program of secondary PCR was the same as the primary PCR, except for the primers (labeled). The products from secondary PCR were run on a 1.0% agarose gel, the bands of proper size (~400 bp) were excised and the DNA were extracted from the bands using QIAEX II gel extraction kit (Qiagen Sciences, MD). Pooled sample containing 25 ng of each purified amplicon was sent to GenomeQuebec (Montreal, QC) for pyrosequencing analysis using 454 Titanium FLX (Roche).

### Sequence processing

The reads were first processed using Quantitative Insights into Microbial Ecology (QIIME) program (1.8.0)[38] to investigate the microbial ecology. 16S rRNA sequencing pre-processing included subsampling at 1000 sequences per sample based on rarefaction analysis. After the primary filtering, 46 debridement samples (Deb) and 51 swab samples (Swab) remained for downstream processing. Operational taxonomic units (OTUs) were assigned based on 97% sequence similarity. The OTUs were assigned taxonomy at the phylum, class, order, family, genus, and species level based on the Greengenes database[39] with the Uclust method. Chimera checking was performed along with the OTU picking step, and singletons were removed simultaneously. The OTU numbers were assigned based on unique OTU reads. OTUs from each sample were normalized to the lowest OTU number identified from the entire sample set for even sampling prior to analyzing the alpha diversity (within sample diversity) and beta diversity (between sample diversity). Shannon index and Simpson index were calculated to indicate alpha diversity through QIIME. Clustering of the obtained profiles was analyzed with PCoA using the UPGMA method, and ANOSIM was conducted to verify the clustering.

The OTUs with relative abundance (RA) > 1% in at least one group of samples were termed major OTUs and further analyzed. The RA of each OTU was compared between sample types (swab or debridement), between diabetes status, before and after treatment, and among wound types. All of the obtained sequences were submitted to NCBI Sequence Read Archive (BioProject ID: PRJNA416137). For wound type analysis, wounds were categorized by anatomical location (foot, leg, Non-F/L) where ‘Non-F/L’ refers to non-lower extremity wounds of acute or surgical origin. Owing to the different progress of each wound, the last visit for each patient was not necessarily the same: some wounds were closed within 1–2 visits and others did not heal until week 4 or later. The comparisons between the first visit (before treatment) and the last visit were conducted to analyze the results of the wound progression and microbiome transition during the study period.

## Results

### Patient demographics and wound scores changes

**Fig 1:**
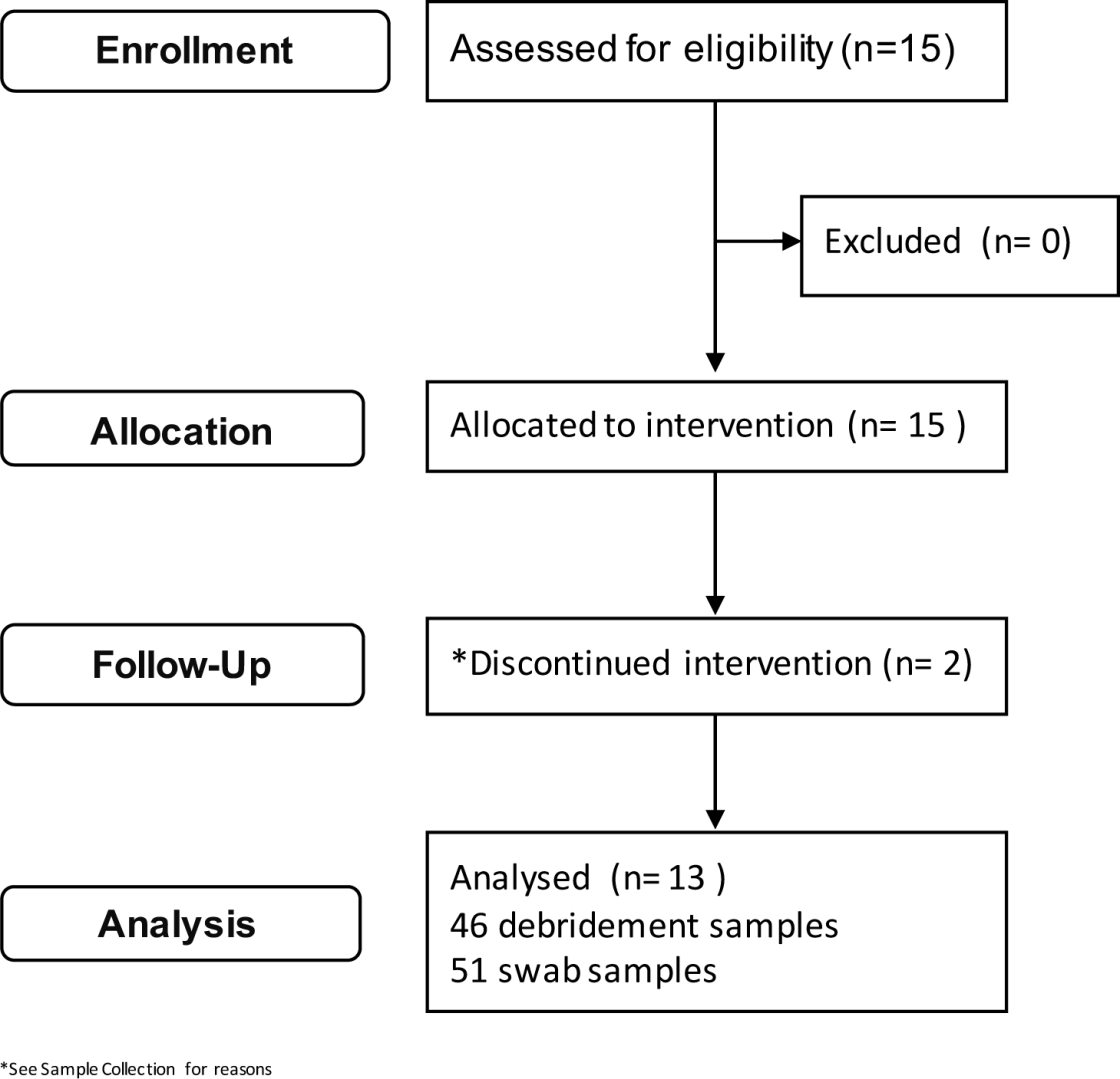
Consort flow-diagram for patient enrollment.

**Fig 2:**
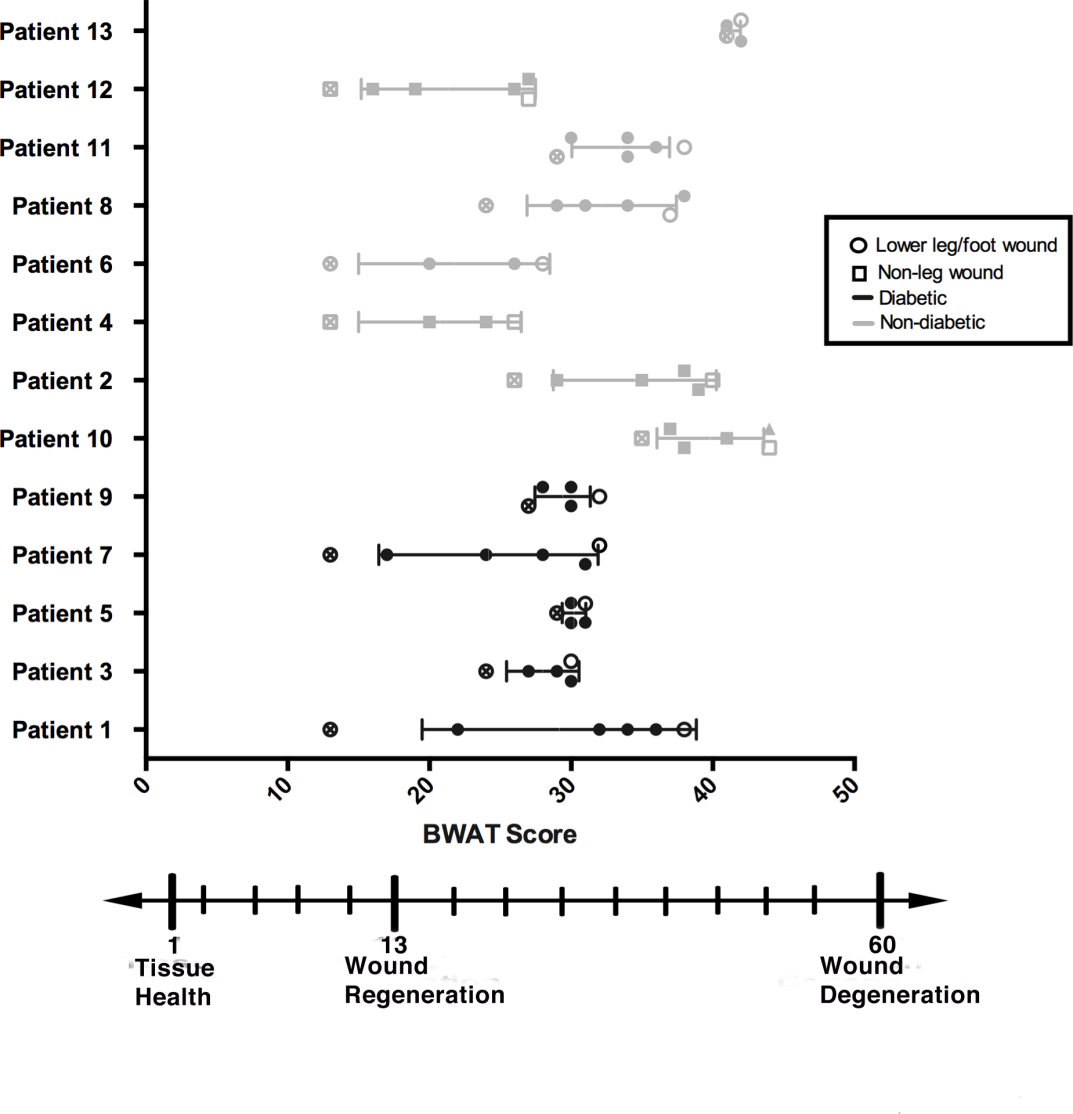
Bates-Jensen Wound Assessment (BWAT) scores of each wound. Patient diabetes status is separated by different colors (black = diabetic, light grey = non-diabetic) and the wound type is separated by shape of data point (square = non-leg, circle = lower leg). Open circle or square indicates Baseline (Visit 1) and crosses indicate the last time point measured (Visit 6). Treatment occurs from visit 2-visit 6 (V2-V6). Solid lines indicate the mean and standard deviation of the BWAT score for each subject timeline.

Patient demographics and wound characteristics are described in Table 1, briefly a cohort of patients with mixed wound etiology were recruited into the study (Fig 1) and co-morbidities that may contribute to healing were recorded. As shown in Fig 2 and described in Table 2, the wound scores determined by the Bates-Jenson Wound Assessment Tool (BWAT)[35] varied among patients with respect to both starting score and extent of progression towards healing. The majority of patients saw substantial improvement in wound score, and full closure was observed in 4 patients. However, in two patients minimal or no wound improvement was observed (Patients 5 and 13).

**Table 2:** Comparison of median wound score (median BWAT ± IQR) between diabetes status and among wound types before and after treatment. Median wound score ratio = visit 6/visit 1 ± IQR, Median wound score change = visit 6—visit 1 ± IQR.

		Median BWAT ± IQR Baseline (V1)	Median BWAT ± IQR (V2-V6)	Median BWAT Ratio ± IQR (V6/V1)	Median BWAT Change ± IQR (V6-V1)
Status	Diabetic (n = 6)	32±5.3	30±7	0.82±0.5	-6±11
	Non-diabetic (n = 7)	37±12	30±11	0.65±0.19	-11±6
Wound Location	Foot ulcer (n = 6)	32±0.75	29±5	0.82±0.18	-5.5±7
	Leg ulcer (n = 3)	38±2	34±9	0.76±0.3	-9±12
	Non-F/L (n = 4)	34±15	29±12	0.62±0.12	-11±2

### Outcomes

#### Swab and debridement samples have distinct microbial communities

**Fig 3:**
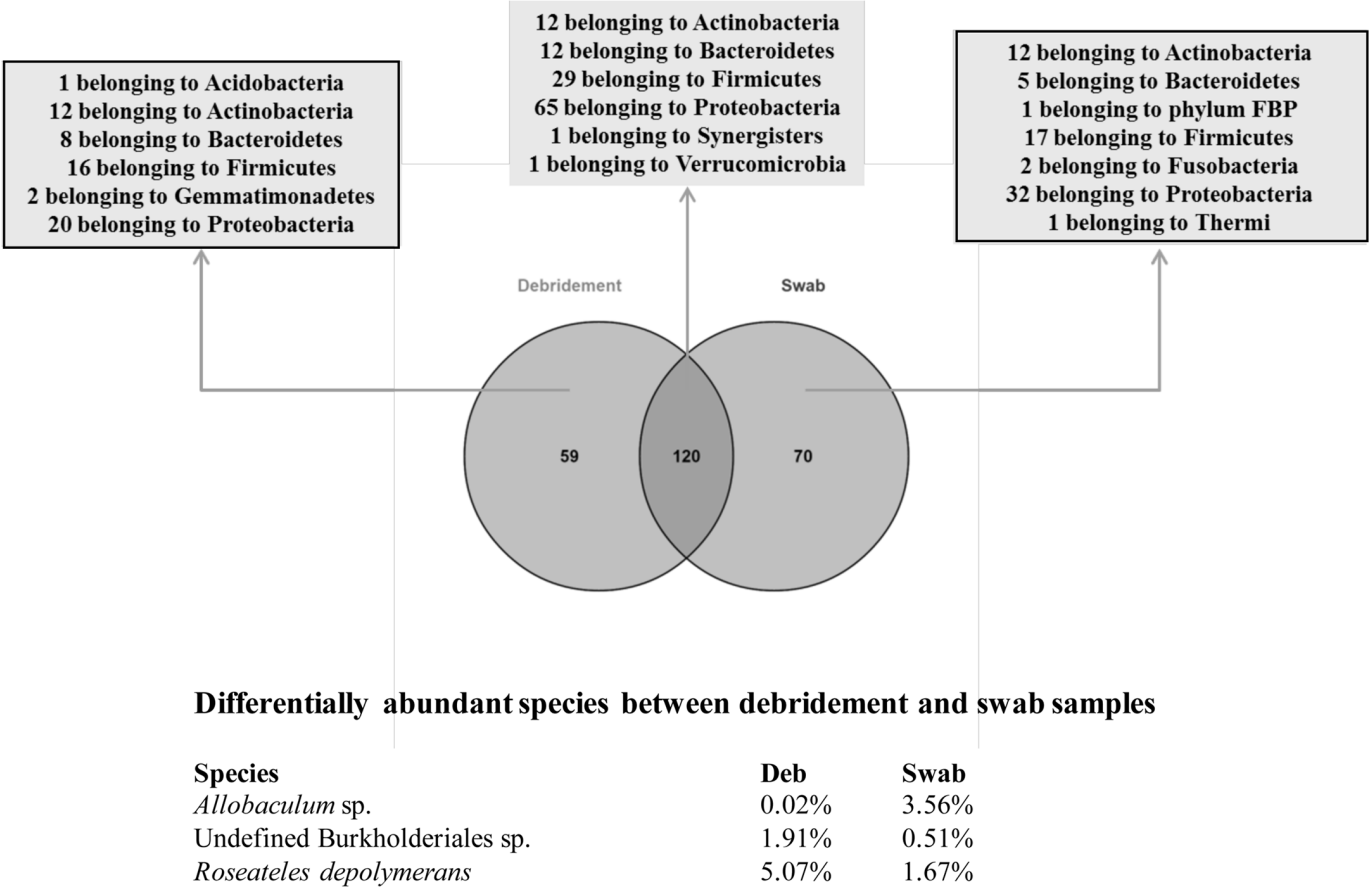
Bacterial communities in swab and debridement samples. The 249 species level OTUs identified from the specimens are classified according to their corresponding phylum. The venn diagram shows 120 OTUs that are found in both debridement and swab specimens, while 59 OTUs are debridement-specific and 70 OTUs are swab-specific (as indicated by the square). OTUs with at least 2-fold numerical differences in relative abundance (>1% for at least one sample type) between the swab and debridement samples. *Allobaculum* sp. is more abundant in swab samples, while an undefined Burkholderiales sp. and *Roseateles depolymerans* are more abundant in debridement samples.

In total, all of the obtained sequences were assigned to 249 distinct OTUs at the species level. The bacterial composition between swab and debridement (deb) samples differed. Among all of the identified OTUs, 120 were observed in both swab and debridement samples, while 59 and 70 OTUs were exclusively found in debridement or swab samples respectively (Fig 3). Although, alpha diversity metrics were similar between sample types (Table A and B in S1 File), we observed unique OTUs belonging to phyla Acidobacteria and Gemmatimonadetes exclusively in debridement samples, while the unique OTUs belonging to phyla FBP, Fusobacteria, and Thermi were exclusively found in swab samples (Fig 3). Among the shared OTUs in sample types, the relative proportions of only 3 OTUs were different between the two sample types (Fig 3): OTUs corresponding to the anaerobic *Allobaculum* sp. was more abundant in swab samples while two aerobic OTUs (*Roseateles depolymerans* and one belonging to the order Burkholderiales) were more abundant in debridement samples.

The bacterial community showed strong patient individual specificity, therefore, the dataset was broken down and analyzed for each patient’s timeline. While there was substantial overlap in community membership (Table C and D in S1 File), the proportion of OTUs differed dramatically between debridement and swab samples in some cases. Sample type-dependent clusters were also found for most of the patients; therefore, swab and debridement samples were analyzed independently for further analyses and discussed further.

#### Common and specialized communities with diabetes status and wound location

To understand whether variation in diabetes status and the wound location impact the wound bacterial community, baseline (V1) samples, collected prior to application of the silver oxysalt dressings, were compared.

**Fig 4:**
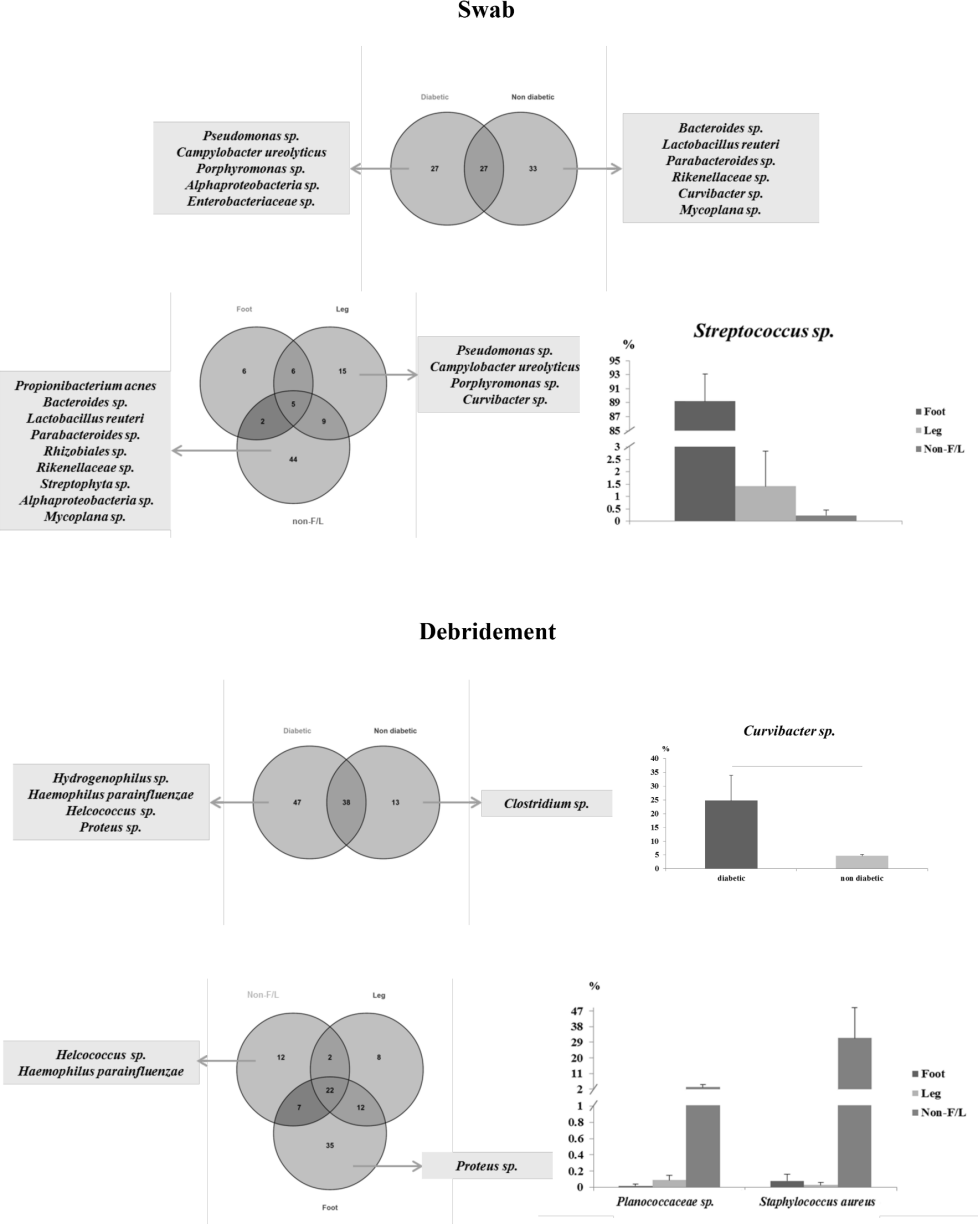
Overview of the bacterial communities between diabetes status and among wound types in pre-treated samples (V1 samples). (A) Plots for swab samples. The Y axis represents the percentage of the OTUs being observed among the total reads. (B) Plots for debridement samples. Y axis represents the percentage of the OTUs being observed among the total reads.

For the swab samples between diabetic and non-diabetic patients, common and unique bacterial OTUs were observed (Fig 4A). Five OTUs were exclusively found in diabetic patients including a *Pseudomonas* sp. and an *Enterobacteriaceae* sp. Six OTUs were only found in non-diabetic patients. Among all of the identified OTUs, relative abundances were comparable between the two diabetic statuses in swab samples. Alpha diversity of the samples did not differ based on diabetes status (Table 3). In contrast, bacterial communities were more disparate between the three wound locations. As shown in Fig 4A, only 25% of the OTUs were shared by all or two of the wound types, while 75% of the OTUs were uniquely present in a single wound type. While certain unique OTUs were found for Leg and Non-F/L wounds respectively, no Foot-specific OTUs were identified in abundance >1% (Fig 4A). Among the more abundant OTUs, *Streptococcus* sp. showed a trend in which it was more abundant in Foot samples than Leg and Non-F/L samples. Wounds located on the foot comprised four diabetic foot ulcers and one anterior toe wound. Observed species and Shannon index were higher for Non-F/L wounds (Table 3).

**Table 3:** Comparison of alpha diversity in pre-treatment (V1) samples.

			observed species median±IQR	Shannon index median±IQR
Swab	Diabetic status	Diabetic (n = 6)	11±9	2.37±1.27
		Non diabetic (n = 7)	13±14	1.83±1.91
	Wound location	Foot ulcer (n = 6)	6±3	0.63±0.26
		Leg ulcer (n = 3)	16±3	1.69±0.72
		Non-F/L (n = 4)	16±7	2.69±0.85
Debridement	Diabetic status	Diabetic (n = 6)	12±11	1.98±1.27
		Non diabetic (n = 7)	14±3	1.84±0.78
	Wound location	Foot ulcer (n = 6)	11±5	1.95±0.62
		Leg ulcer (n = 3)	12±8	1.53±0.45
		Non-F/L (n = 4)	14±3	2.45±0.39

For the debridement samples between diabetic and non-diabetic patients, common and diabetic-specific OTUs were also observed (Fig 4B). Four OTUs were associated with diabetic patients and one OTU was associated with non-diabetic patients. The relative abundance of *Curvibacter* sp. appeared to be higher in diabetic patients compared to non-diabetic patients (Fig 4B). The alpha diversity metrics were not different between diabetes status (Table 3). Among the three body locations, two OTUs were uniquely Non-F/L associated and one OTU was foot associated. Proportions of undefined *Planococcaceae* sp. and *Staphylococcus aureus* were higher in Non-F/L wounds than that of Foot or Leg wounds (Fig 4B). The alpha diversity for the samples did not differ among wound location (Table 3).

#### Temporal changes in microbial communities

**Fig 5:**
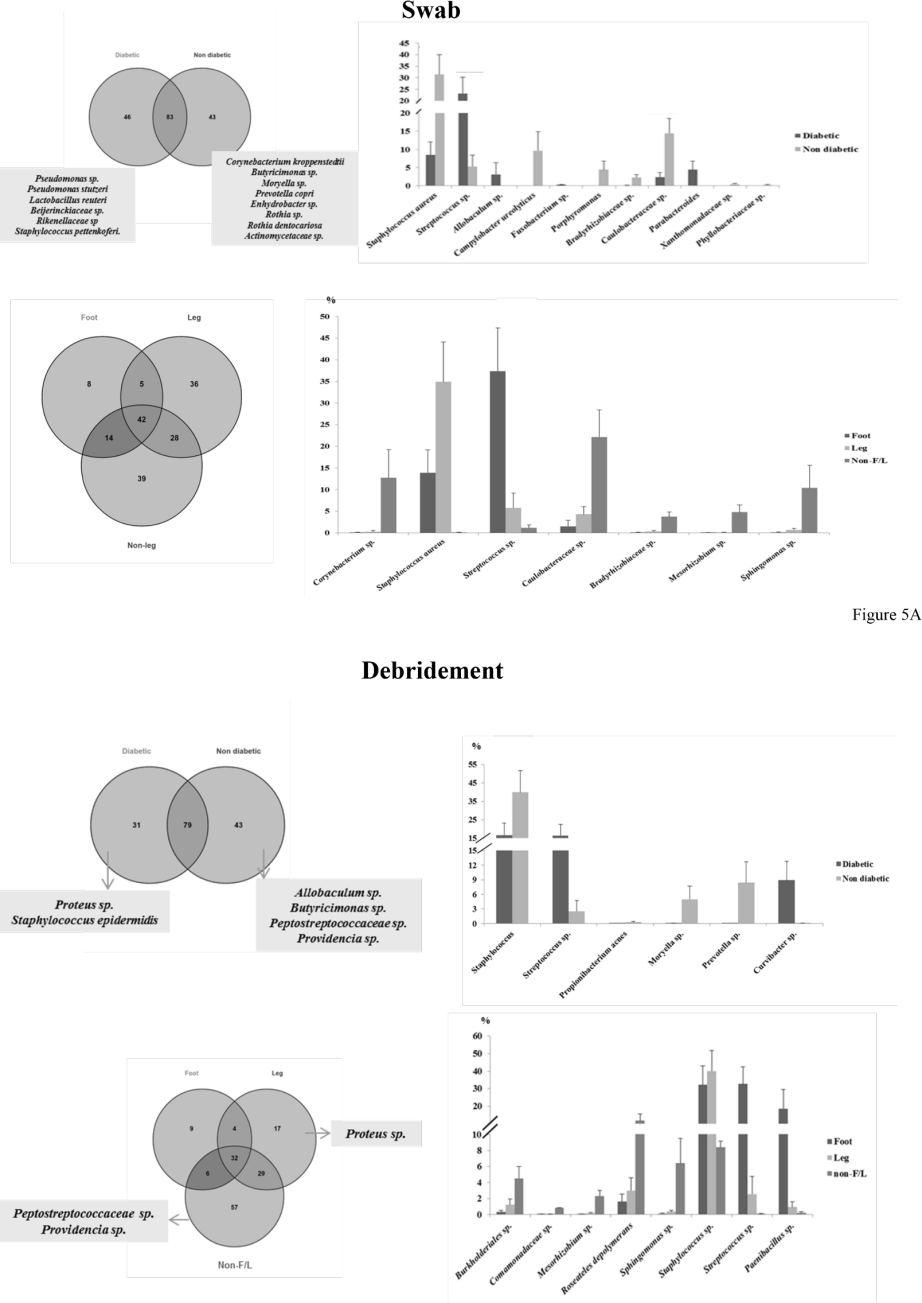
PCoA clustering, common/unique bacterial OTUs at species level, and differentially abundant bacterial OTUs between diabetes status and among wound types in post-treatment samples (V2-V6 samples). (A) Plots for swab samples. Venn diagrams suggest that diabetic status-associated and wound type-associated OTUs exist in swab samples. Differentially abundant OTUs are listed in the bar chart. (B) Plots for debridement samples. Venn diagrams suggest that diabetic status-associated and wound type-associated OTUs exist in debridement samples. Differentially abundant OTUs are listed in the bar chart.

The bacterial communities of the samples collected after initial wound dressing application were then compared between patient diabetes status and among wound location. In both the swab samples and debridement samples, bacterial profiles did not form obvious clusters according to diabetes status or wound location. Similar trends as the pre-treatment samples were observed, that some OTUs were commonly shared by diabetes status and by the three wound types, while some OTUs were diabetes-specific or wound location-specific (Fig 5A and 5B). The most abundant OTUs in the entire dataset did not change during antimicrobial treatment (data not shown), but the diabetes status-specific or wound location-specific OTUs were shifted after the dressing was applied (Figs 4 and 5). The proportion of the OTUs that occupied different proportions between diabetes status and among wound locations for both swab and debridement samples are listed in Fig 5. The relative proportions of *Staphylococcus* sp. shifted from pre-treatment samples, increasing in abundance in foot and leg wounds for both swab and debridement samples but markedly decreasing in non-F/L wounds. For both swab and debridement samples, alpha diversity and wound score changes did not differ between diabetes status, while among the three wound locations, non-leg wounds displayed a more diverse microbiome than foot and leg ulcers (Table 4).

**Table 4:** Comparison of alpha diversity in treated (V2-V6) samples.

			observed species median±IQR	Shannon index median±IQR
Swab	Diabetic status	Diabetic (n = 6)	13±9	2.37±1.27
		Non diabetic (n = 7)	13±14	1.83±1.91
	Wound location	Foot ulcer (n = 6)	10±6	1.49±1.43
		Leg ulcer (n = 3)	11±7	1.83±1.62
		Non-F/L (n = 4)	27±11	3.11±0.66
Debridement	Diabetic status	Diabetic (n = 6)	12±10	2.02±1.00
		Non diabetic (n = 7)	11±11	1.45±1.50
	Wound location	Foot ulcer (n = 6)	9±3	1.74±0.97
		Leg ulcer (n = 3)	10±6	1.45±0.95
		Non-F/L (n = 4)	24±10	2.85±0.69

#### Responses of the microbial communities to topical antimicrobial treatment

As the bacterial community of each patient varied among each other, a comparison of the bacterial communities before (V1) and after (all later visits) the silver wound dressing was applied was conducted for the samples collected from each patient respectively. The bacterial OTUs that appeared or disappeared after the wound dressing treatment differed for each individual, and some bacterial OTUs showed different trends for different sample types and/or for different patients (Table C and D in S1 File).

**Fig 6:**
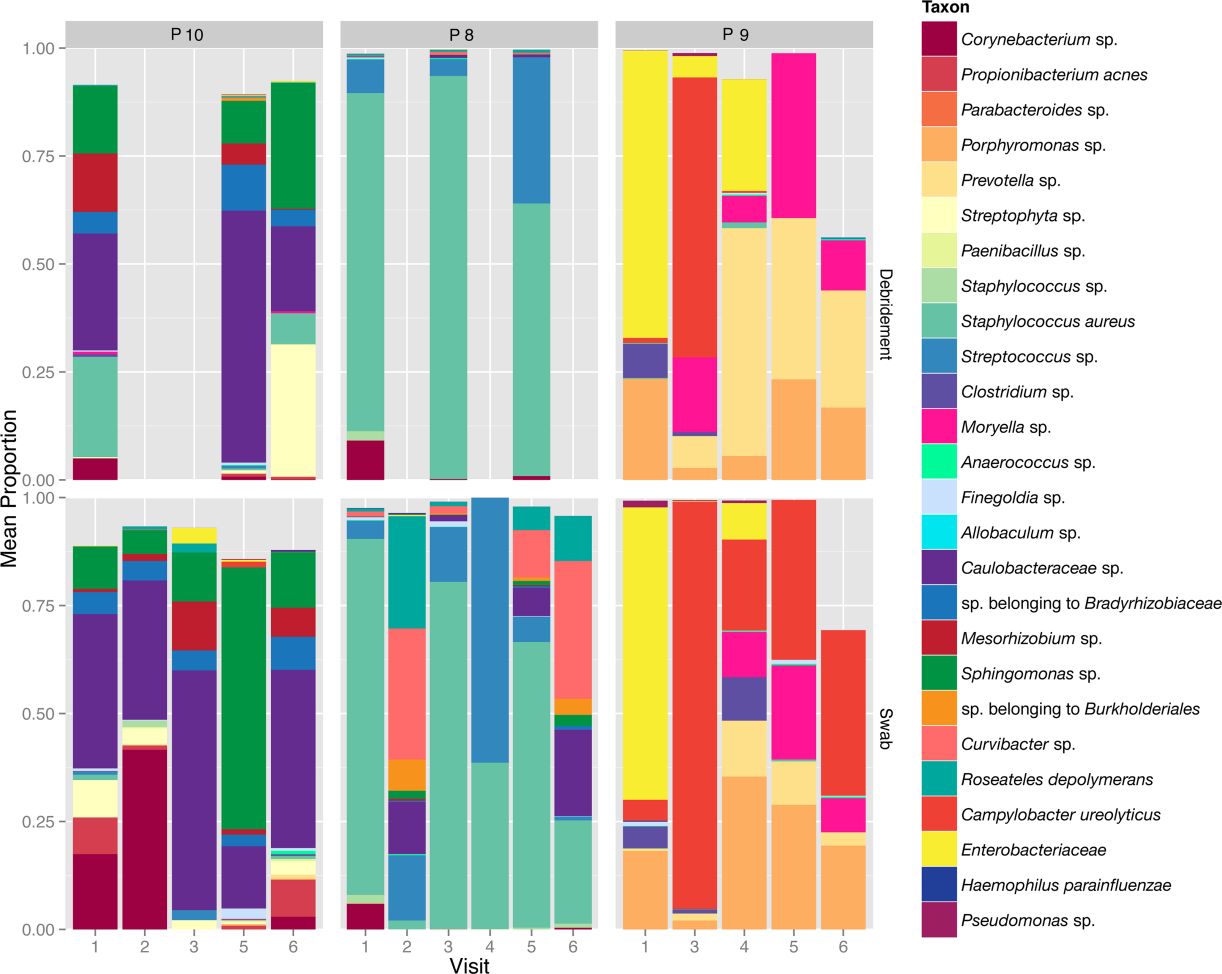
Bacterial relative abundance (>1% abundance in all samples) plots for three patient timelines. Plots are split by debridement samples (top panel) and swab samples (bottom panel). Wound locations are as follows: P10 (post-surgical abdomen, non-F/L wound), P8 (venous leg ulcer), P9 (diabetic foot ulcer). Debridement samples were not taken on visit 2.

To determine if the alpha diversity or within sample diversity changed over time, the ratio (last visit:first visit) and the change (last visit minus first visit) of the Shannon index (which takes into account the total number of OTUs and evenness in each sample) for the communities were calculated for each patient between the first visit (V1) and the last visit. We found that neither diabetes status nor wound type influenced the Shannon index changes. The bacterial communities in each sample did change over time where three individual patient timelines from the three body locations are shown in Fig 6, demonstrating differences between patients, sample type, and over time.

## Discussion

To our knowledge this is the first longitudinal survey of bacterial communities in non-healing chronic wounds during the course of treatment with a topical antimicrobial silver dressing. This study included 13 wounds of varying etiology (9 lower leg, 5 Non F/L) with 46% of the population diabetic. Our data indicate that the microbiota of the wound bed differs in composition by body site and wound type. As a result, the mixed etiologies lead to limitations in data interpretation related to outcomes, particularly the sample size is too small and variable for statistical analysis. However, the observations reported here provide a basis for assessment in a larger sample size of single etiology or disease state.

Each wound was defined as non-healing or stalled if greater than six weeks in duration. 9 of 13 wounds were dressed with another topical antimicrobial dressing prior to enrollment in the study (8 with silver dressings, 1 with a Gentian violet/methylene blue dressing). 2 of 13 wounds had little or no improvement (P5 and 13) and in both cases clinical factors could be attributed to continued chronicity. In P5 foot architecture impacted gait and risk of infection, leading to the treatment goal of infection prevention until surgical intervention could correct the problem. P13 presented with complex chronic venous insufficiency and inadequate edema management that was not resolved for an additional 11 months post-study. In both of these wounds the microbiota was less diverse, less dynamic and dominated by *Staphylococcus aureus*. P5 also had a high abundance of *Streptococcus* sp, a bacterium associated with the skin and pathogenesis[40–42]. 8 of 13 wounds had a reduction in size > 75% within the four-week study period and 11 of 13 wounds were responding towards closure. Each patient with a wound progressing towards healing was distinct in their wound microbiome from each other, such that the population size was not large enough for predictive or prognostic modelling to identify OTUs that contribute to outcomes.

We set out to evaluate if the microbiome can be measured while an antimicrobial dressing is in use because it is presumed that topical antimicrobials act by disruption of microbial colonization within the wound bed. This is relevant because critical colonization and establishment of a biofilm is hypothesized to be linked to stalled healing and chronic inflammation[43]. A large proportion of the cohort appeared to move from a non-healing state towards a healing state, indicating that the silver oxynitrate dressing may promote an environment to facilitate wound closure in combination with good clinical care. Due to the individuality of the samples it was difficult to measure global targets and efficacy of the wound dressing. Further, the composition of the microbial community was measured at all time points suggesting that the dressing does not completely reduce the bioburden in the wound, however the overall quantitative bioburden measurement was outside the scope of this study and would strengthen future works. In individual patient timelines we did observe instances of fluctuation in the bacterial community structure after the antimicrobial dressing was applied. In P8, *Staphylococcus aureus* is reduced from >80% of the community to < 20% in the first 24 hrs and the community structure remains dynamic as the wound is closing (Fig 6).

Diabetes status did not appear to influence healing rates which supports recent studies reporting subtle but not significant differences between diabetic foot and healthy skin proliferative ability[44]. While our study was not sufficiently powered to distinguish between diabetic and non-diabetic wounds, it does support implementation of standard wound management that will result in better outcomes and prevent further complications. This includes unresolved infection that could lead to limb amputation regardless of diabetes status. In this study all wounds were sharp debrided and thoroughly cleansed prior to dressing application, which represents a treatment modality critical to healthy wound bed preparation[45] but remains inconsistent amongst practitioners. In future studies the effects of serial debridement alone and in combination with an effective topical antimicrobial dressing would provide interesting evidence on the control of bioburden and influence on microbial composition, a limitation to the current study. Indeed, our results support the hypothesis that both host factors and choice of intervention contribute towards healing and should be considered for personalized treatment plans.

It is clear from our data that there are distinct differences between lower extremity wounds–leg or foot ulcers–and wounds occurring as a result of post-surgical complication, skin tears or trauma to other parts of the body. Wound progression and microbial composition varies amongst the two groupings. This is also consistent with healthy skin microbiota, harboring distinct communities across diverse body sites of varying moisture and other micro-environment content[46–49]. This is an important observation that suggests chronic wound microbiomes differ depending on body site location and thus may require different strategies for treatment and management of microbial bioburden depending on the microbiome composition.

Wound progression and healing have been related to the bacteria within a wound environment[50]. Previous knowledge has been limiting as many studies of the wound microbiota largely relied on culture-based methods, resulting in a large proportion microbes not revealed because they do not grow in standard clinical microbiology protocols. Therefore, the current study attempted to look in-depth at the wound microbiome and assess if it can be measured over time when topical antimicrobial (silver oxynitrate) wound dressing treatment is applied. It further aimed to compare the microbiome composition with different sampling methodology and wound types (or body site). To the best of our knowledge, this is the first time the microbiome between swab and debridement samples has been reported (Fig 3), suggesting that the bacterial communities between the wound bed tissue and slough or eschar on the surface differ. The following analyses for each wound (Table C and D in S1 File) further supported that in samples collected from the same wound, different bacterial species occupied different niches as per their substrate/environment preferences. This finding supports clinical best practices pertaining to good wound bed preparation, as well as cleansing and sampling techniques for the clinical microbiology laboratory. This is because a surface swab prior to debridement and cleansing is not indicative of bacterial species found deeper within the wound bed.

The bacterial communities of the samples collected before application of the silver dressing (V1 samples) suggests that there is not a core microbiota for a particular sample type, or to patient diabetes status, or to wound type (Table 3 and Fig 4), although our study was not powered for determining statistical significance. Prior to the wound dressing application, the relative abundance of the majority of the predominant OTUs did not differ between diabetic status or among wound locations for both swab and debridement samples, however in a larger cohort statistical difference may be detectable.

As it was previously reported that the microbiome was different between diabetic status and wound locations, analyses were then conducted on V2-V6 samples to identify whether commonality exists depending on patient diabetes status and wound types after treatment. Patient diabetes status did not influence the wound microbiome in terms of sample alpha diversity and for the presence of each OTU after wound dressing treatment (Table 4), indicating that the wound dressing applied in the current study did not trigger different responses according to patient diabetes status. However, the relative abundance of *Staphylococcus* sp./*Staphylococcus aureus* appeared to be higher in non-diabetic patients while *Streptococcus* sp. was higher in diabetic patients for both swab and debridement samples (Fig 5). If the metabolic profiles of the blood and skin between diabetic and non-diabetic patients differs and influences bacterial colonization, the microbial responses to antimicrobial treatments may also differ. Further analyses on the metabolome of blood and skin samples and their associations with the microbiome would provide better insights into microbial communities and their prevalence between diabetic and non-diabetic patients.

More variation was detected when samples were compared among wound locations. In addition to the numerous wound-specific OTUs and varied alpha diversities (Table 4) being observed among the three wound locations (Fig 5), the relative abundance of certain OTUs was also different among the three wound locations (Table 4). It is noticeable that among these unique OTUs classified, *Staphylococcus aureus*, and *Streptococcus* sp. are the most abundant OTUs being identified in almost all foot/leg wounds, while an undefined Caulobacteraceae sp. is one of the main classified OTUs in almost all Non-F/L wound types (data not shown), suggesting that these OTUs may be of different importance in different body sites and among different wound types. This was consistent with Redel et al.[51] that the microbiome of different wound locations are comprised of predominant species. *Staphylococcus aureus*, and *Streptococcus* sp. are among the main pathogens widely reported from previous wound studies[52,53], but the current study did not have enough data to support a hypotheses on the influence of these OTUs in the related wound types and how they actually react to the dressing or impact healing. *In vitro* studies show that Ag oxynitrate has high antibacterial activity against both species in pure culture (data not shown) but this study supports the lack of correlation between *in vitro* testing and *in situ* activity. Future studies focusing on these common pathogens could measure the transcriptome and biochemistry of the wound bacteria to better understand their functional role in delayed healing. Conversely, Caulobacteraceae was reported recently from a 29-year-old male with history of hypertension and morbid obesity presented with bilateral lower extremity swelling and was admitted for cellulitis in the leg (https://medicine.wright.edu/sites/default/files/page/attachments/IDNewsletter_2015_02.pdf). According to that report, this species was recently separated from its original classification of a *Pseudomonas* species, and its biochemistry and pathogenic features have not been well defined. Based on these results, further investigation of how each organism functions in different wound types would be valuable to personalize wound treatment.

To understand how each OTU changes during the wound progression is also important for supplying information to develop effective wound care strategies. However, owing to a small number of samples, and the small proportion of most of the identified OTUs, the bacteria being depleted or promoted after wound dressing treatment (Table C and D in S1 File) should also be considered. It is difficult to distinguish if all of these OTUs are more sensitive to the silver dressing compared to the unchanged ones, or if they are changing in response to a more favorable healing environment as evidenced by improvement in wound scores and progression. These apparently more vulnerable organisms merit further study as potential targets in chronic wounds. This study was not without limitations and the authors feel it would be strengthened from a survey of the peri-wound and healthy surrounding skin microbiota, particularly to evaluate if wound closure results in restoration of a similar microbiota. Quantification of bacterial burden would also provide insight into the efficacy of the topical antimicrobial’s ability to reduce overall bioburden in the wound.

In conclusion, in this pilot study we determined that it is feasible to measure the microbiome of chronic wounds when dressed with a topical antimicrobial agent. Our results suggest that future studies would benefit from including only a single etiology due to difference observed in the microbiome between wound types. We could not identify a clear trend in response to the antimicrobial wound dressing treatment for particular OTUs even though *in vitro* susceptibility testing would predict broad spectrum activity. The clear separation of microbiome between swab and debridement samples also suggests that sampling is important. Standard wound protocols should consider the complexity of the wound environment and diversity of the microbiota across space and time.

## Supporting information

S1 File Supplementary tables. (PDF)

S2 File IRB approved protocol. (PDF)

S3 File Thabane checklist. (XLSX)

S4 File TREND statement and checklist. (PDF)
