# Supplementary material for: Measuring the microbiome of chronic wounds with use of a topical antimicrobial dressing – A feasibility study
Source: PLoS One. 2017 Nov 20;12(11):e0187728. doi: 10.1371/journal.pone.0187728 (PMC5695787; doi:10.1371/journal.pone.0187728)
Supplement: S1 File — (PDF) [file pone.0187728.s001.pdf]

Table A. Alpha diversity of each swab sample.

Sample	Patient	Visit	Disease	Wound	Chao1	Goods coverage	Observed species	Shannon	Simpson
S1-2	1	2	diabetic	leg	28	0.976	15	2.39	0.73
S1-3	1	3	diabetic	leg	20	0.978	14	1.77	0.52
S1-6	1	6	diabetic	leg	51	0.946	27	3.33	0.84
S2-1	2	1	non diabetic	Non-F/L	30	0.973	16	2.63	0.76
S2-5	2	5	non diabetic	Non-F/L	37	0.957	24	3.30	0.85
S2-6	2	6	non diabetic	Non-F/L	16	0.978	11	0.93	0.25
S3-1	3	1	diabetic	foot	4	0.995	3	0.40	0.13
S3-2	3	2	diabetic	foot	4	0.995	3	0.69	0.28
S3-3	3	3	diabetic	foot	18	0.979	14	1.54	0.45
S3-6	3	6	diabetic	foot	10	0.990	7	1.49	0.57
S4-2	4	2	non diabetic	Non-F/L	38	0.965	22	2.88	0.74
S5-1	5	1	diabetic	foot	13	0.984	10	0.95	0.27
S5-2	5	2	diabetic	foot	9	0.990	7	1.48	0.58
S5-3	5	3	diabetic	foot	12	0.983	8	1.35	0.54
S5-4	5	4	diabetic	foot	17	0.983	14	2.05	0.63
S5-5	5	5	diabetic	foot	16	0.980	10	0.77	0.21
S5-6	5	6	diabetic	foot	7	0.990	5	0.40	0.11
S6-3	6	3	non diabetic	foot	6	0.991	5	0.21	0.05
S6-4	6	4	non diabetic	foot	11	0.989	9	1.00	0.29
S7-2	7	2	diabetic	foot	12	0.988	11	2.28	0.73
S7-3	7	3	diabetic	foot	39	0.955	20	2.43	0.72
S7-4	7	4	diabetic	foot	23	0.973	17	2.71	0.80
S7-5	7	5	diabetic	foot	23	0.979	17	2.99	0.84
S8-1	8	1	non diabetic	leg	25	0.966	19	1.44	0.35
S8-2	8	2	non diabetic	leg	26	0.975	16	2.71	0.80
S8-3	8	3	non diabetic	leg	14	0.982	11	1.14	0.35
S8-4	8	4	non diabetic	leg	3	0.996	3	0.98	0.47
S8-5	8	5	non diabetic	leg	20	0.978	13	1.88	0.55
S8-6	8	6	non diabetic	leg	23	0.974	17	2.68	0.79
S9-1	9	1	diabetic	leg	16	0.983	13	1.80	0.55
S9-3	9	3	diabetic	leg	8	0.991	7	0.42	0.10
S9-4	9	4	diabetic	leg	20	0.981	12	2.61	0.80
S9-5	9	5	diabetic	leg	14	0.987	11	2.37	0.76
S9-6	9	6	diabetic	leg	18	0.984	15	2.88	0.80
S10-1	10	1	diabetic	Non-F/L	35	0.961	28	3.44	0.84
S10-2	10	2	diabetic	Non-F/L	38	0.954	28	2.93	0.76
S10-3	10	3	diabetic	Non-F/L	19	0.988	17	2.41	0.66
S10-5	10	5	diabetic	Non-F/L	63	0.926	36	3.16	0.75
S10-6	10	6	diabetic	Non-F/L	55	0.935	33	3.28	0.79
S11-3	11	3	non diabetic	leg	4	0.995	4	0.76	0.32
S11-4	11	4	non diabetic	leg	19	0.978	8	0.95	0.35
S11-6	11	6	non diabetic	leg	37	0.961	25	3.05	0.79
S12-1	12	1	non diabetic	Non-F/L	25	0.970	17	1.84	0.52
S12-2	12	2	non diabetic	Non-F/L	40	0.954	25	2.78	0.72
S12-4	12	4	non diabetic	Non-F/L	45	0.956	33	3.68	0.86
S12-5	12	5	non diabetic	Non-F/L	56	0.936	36	3.78	0.86
S12-6	12	6	non diabetic	Non-F/L	44	0.956	37	4.21	0.92
S13-1	13	1	non diabetic	leg	25	0.976	17	2.88	0.81
S13-3	13	3	non diabetic	leg	9	0.986	6	0.26	0.06
S13-4	13	4	non diabetic	leg	14	0.984	11	1.68	0.60
S13-6	13	6	non diabetic	leg	12	0.989	10	1.73	0.54

Table B. Alpha diversity of each debridement sample.

Sample	Patient	Visit	Disease	Wound	Chao1	Goods coverage	Observed species	Shannon	simpson
D1-1	1	1	diabetic	leg	13	0.981	10	1.790	0.581
D1-3	1	3	diabetic	leg	23	0.962	16	2.595	0.760
D1-4	1	4	diabetic	leg	15	0.976	11	1.413	0.425
D1-5	1	5	diabetic	leg	10	0.985	8	1.187	0.359
D1-6	1	6	diabetic	leg	39	0.943	20	2.791	0.774
D2-1	2	1	non diabetic	Non-F/L	16	0.977	13	2.405	0.745
D2-3	2	3	non diabetic	Non-F/L	13	0.979	10	2.110	0.707
D2-4	2	4	non diabetic	Non-F/L	31	0.958	14	2.289	0.715
D2-5	2	5	non diabetic	Non-F/L	42	0.949	20	2.974	0.806
D2-6	2	6	non diabetic	Non-F/L	20	0.973	11	2.202	0.711
D3-1	3	1	diabetic	foot	52	0.922	25	3.093	0.822
D3-3	3	3	diabetic	foot	12	0.983	10	1.931	0.613
D3-4	3	4	diabetic	foot	10	0.988	8	1.718	0.617
D3-6	3	6	diabetic	foot	10	0.985	7	1.176	0.438
D4-1	4	1	non diabetic	Non-F/L	21	0.968	14	2.465	0.753
D4-3	4	3	non diabetic	Non-F/L	39	0.935	24	2.892	0.769
D5-1	5	1	diabetic	foot	12	0.983	10	1.456	0.450
D5-3	5	3	diabetic	foot	8	0.985	5	0.947	0.376
D5-4	5	4	diabetic	foot	22	0.966	15	2.300	0.661
D5-5	5	5	diabetic	foot	9	0.990	8	1.986	0.676
D5-6	5	6	diabetic	foot	9	0.983	6	0.586	0.171
D6-1	6	1	non diabetic	foot	13	0.983	9	1.780	0.598
D6-4	6	4	non diabetic	foot	13	0.981	9	0.989	0.268
D7-1	7	1	diabetic	foot	13	0.990	11	2.106	0.605
D7-3	7	3	diabetic	foot	26	0.973	16	3.030	0.820
D8-1	8	1	non diabetic	leg	16	0.976	13	1.558	0.432
D8-3	8	3	non diabetic	leg	9	0.983	7	0.522	0.139
D8-5	8	5	non diabetic	leg	10	0.982	7	1.223	0.490
D9-1	9	1	diabetic	leg	8	0.990	7	1.459	0.526
D9-3	9	3	diabetic	leg	9	0.992	9	1.733	0.551
D9-4	9	4	diabetic	leg	19	0.977	14	2.244	0.666
D9-5	9	5	diabetic	leg	8	0.995	8	2.020	0.700
D9-6	9	6	diabetic	leg	22	0.976	14	3.085	0.854
D10-1	10	1	diabetic	Non-F/L	33	0.951	24	3.316	0.844
D10-5	10	5	diabetic	Non-F/L	49	0.924	25	2.618	0.661
D10-6	10	6	diabetic	Non-F/L	39	0.937	22	2.970	0.809
D11-1	11	1	non diabetic	leg	10	0.982	6	0.829	0.312
D11-3	11	3	non diabetic	leg	6	0.990	4	0.761	0.303
D11-4	11	4	non diabetic	leg	11	0.983	9	1.438	0.469
D11-6	11	6	non diabetic	leg	17	0.967	11	1.147	0.335
D12-1	12	1	non diabetic	Non-F/L	20	0.970	13	1.899	0.551
D12-4	12	4	non diabetic	Non-F/L	57	0.899	34	3.357	0.783
D12-6	12	6	non diabetic	Non-F/L	58	0.906	35	3.832	0.876
D13-1	13	1	non diabetic	leg	24	0.966	15	2.646	0.795
D13-3	13	3	non diabetic	leg	16	0.970	11	1.466	0.504
D13-4	13	4	non diabetic	leg	5	0.990	3	0.135	0.032

Table C. Bacterial phylotype changes in swab samples after wound dressing treatment.

	disappear	appear
P1	-	-
P2	*Corynebacterium* sp.<br>*Lactobacillus* sp.<br>Clostridiales sp.<br>*Blautia* sp.<br>*Clostridium difficile*<br>*Anaerococcus* sp.<br>*Finegoldia* sp.<br>*Acidocella* sp.<br>*Proteus* sp.<br>*Halomonas* sp.<br>*Pasteurella* sp.	*Streptococcus* sp.<br>Rickettsiales sp.
P3	*Rhodococcus fascians*<br>*Staphylococcus* sp.<br>*Staphylococcus pettenkoferi*<br>Gemellales sp.	*Actinomyces* sp.<br>*Corynebacterium* sp.<br>*Dermabacter* sp.<br>Planococcaceae sp.<br>*Peptoniphilus sp.*
P4	-	-
P5	Clostridiaceae sp.<br>*Ruminococcus flavefaciens*<br>*Allobaculum* sp.<br>*Fusobacterium* sp.<br>*Methylobacterium komagatae*	Actinomycetales sp.<br>*Pseudoclavibacter bifida*<br>*Staphylococcus haemolyticus*<br>Caulobacteraceae sp.<br>Burkholderiales sp.<br>Oxalobacteraceae sp.<br>*Stenotrophomonas geniculate*<br>*Akkermansia muciniphila*
P6	-	-
P7	-	-
P8	*Corynebacterium simulans*<br>*Bacillus* sp.<br>*Paenibacillus* sp.<br>Planococcaceae sp.<br>*Staphylococcus haemolyticus*<br>*Staphylococcus pettenkoferi*<br>*Alloiococcus otitis*<br>*Ruminococcus gnavus*<br>*Anaerococcus* sp.<br>*Peptoniphilus* sp.<br>*Haemophilus parainfluenzae*	Gemellales sp.<br>*Ruminococcus* sp.<br>Bradyrhizobiaceae sp.<br>Sphingomonadaceae sp.<br>*Sphingobium* sp.<br>*Salinivibrio costicola*
P9	*Clostridium* sp.<br>Caulobacteraceae sp.	*Bacteroides* sp.<br>*Bacteroides fragilis*

	Bradyrhizobiaceae sp.	*Butyricimonas* sp.
	Sphingomonas sp.	*Moryella* sp.
	Enterobacteriaceae sp.	Peptostreptococcaceae sp.
	Enterobacter ludwigii	*Dialister* sp.
	Pseudomonadaceae sp.	Clostridiales sp.
	Providencia sp.	1-68 sp.
	Pseudomonas sp.	*Peptoniphilus* sp.
P10	*Rothia dentocariosa*	Micrococcaceae sp.
	Rothia mucilaginosa	*Rothia* sp.
	Streptococcus sp.	*Friedmanniella* sp.
	Alphaproteobacteria sp.	*Porphyromonas* sp.
	Arthrospira sp.	*Prevotella* sp.
	Ellin329 sp.	*Paenibacillus* sp.
	Rhodoplanes sp.	Gemellaceae sp.
	Rickettsiales sp.	Lactobacillales sp.
	Novosphingobium sp.	Clostridiaceae sp.
	Sphingomonas wittichii	*Anaerococcus* sp.
	Rhodocyclaceae sp.	*Fusobacterium* sp.
		Caulobacter sp.
		Rhizobiaceae sp.
		Rhizobium sp.
		Curvibacter sp.
		Haemophilus parainfluenzae
		Acinetobacter lwoffii
		Pseudomonas sp.
P12	-	-
P13	-	-
P14	*Knoellia subterranean*	Actinomyces sp.
	Rothia dentocariosa	*Nocardiopsis exhalans*
	Mycobacterium llatzerense	*Propionibacterium* sp.
	Bacteroides uniformis	*Bacteroides* sp.
	Planococcaceae sp.	*Bacteroides fragilis*
	Brevundimonas poindexterae	*Porphyromonas* sp.
	Rhodobacteraceae sp.	*Butyricimonas* sp.
	Sphingopyxis sp.	*Bacillus* sp.
	Burkholderiaceae sp.	*Paenibacillus* sp.
		Planococcus maitriensis
		Staphylococcus aureus
		Staphylococcus epidermidis
		Lactobacillales sp.
		Streptococcus sp.
		Caloramator sp.
		Moryella sp.
		Anaerococcus sp.
		Finegoldia sp.
		Peptoniphilus sp.
		Allobaculum sp.

		Gemmatimonas sp.<br>*Phenylobacterium* sp.<br>Ellin329 sp.<br>*Balneimonas* sp.<br>*Methylobacterium* sp.<br>Rhizobiaceae sp.<br>*Paracoccus aminovorans*<br>*Novosphingobium* sp.<br>*Sphingomonas asaccharolytica*<br>Burkholderiales sp.<br>Alcaligenaceae sp.<br>*Sutterella* sp.<br>Oxalobacteraceae sp.<br>Rhodocyclaceae sp.<br>*Campylobacter* sp.<br>Shewanellaceae sp.<br>*Ewingella Americana*<br>Moraxellaceae sp.<br>*Acinetobacter* sp.<br>*Psychrobacter pulmonis*<br>*Pseudomonas* sp.<br>Xanthomonadaceae sp.
P15	*Corynebacterium* sp.<br>Caulobacteraceae sp.<br>*Mesorhizobium* sp.<br>*Sphingomonas* sp.	*Dermabacter* sp.<br>Gemellales sp.

Table D. Bacterial phylotype changes in debridement samples after wound dressing treatment.

	disappear	appear
P1	*Streptococcus* sp.	*Parabacteroides distasonis*
	Paracoccus aminovorans	Rikenellaceae sp.
	Burkholderia sp.	S24-7 sp.
	Variovorax paradoxus	Planococcaceae sp.
	Ralstonia sp.	*Staphylococcus aureus*
		Staphylococcus haemolyticus
		Arthrospira sp.
		Mycoplana sp.
		Rhizobiales sp.
		Methylobacteriaceae sp.
		Sphingomonadales sp.
		Sphingomonadaceae sp.
		Sutterella sp.
		Oxalobacteraceae sp.
		Spirobacillales sp.
		Salinivibrio costicola
		Dyella sp.
		Akkermansia muciniphila
P2	*Corynebacterium* sp.	*Pedobacter* sp.
	Bacteroides sp.	*Enterococcus* sp.
	Anaerococcus sp.	*Ruminococcus* sp.
	Finegoldia sp.	*Mycoplana* sp.
	Peptoniphilus sp.	*Paracoccus* sp.
	Proteus sp.	Rhodospirillaceae sp.
	Pseudoalteromonas porphyrae	Sphingomonadaceae sp.
		Burkholderiaceae sp.
		Oxalobacteraceae sp.
		Ralstonia sp.
		Pseudomonas stutzeri
P3	Acidimicrobiales sp.	*Staphylococcus epidermidis*
	Corynebacterium sp.	*Peptoniphilus* sp.
	Microbacteriaceae sp.	
	Micrococcaceae sp.	
	Mycobacterium llatzerense	
	Rhodococcus fascians	
	Nocardioidaceae sp.	
	Propionibacteriaceae sp.	
	Propionibacterium acnes	
	Bacteroides acidifaciens	
	Porphyromonas sp.	
	Prevotella sp.	
	Rikenellaceae sp.	
	S24-7 sp.	
	Gillisia sp.	
	Cloacibacterium sp.	
	Streptophyta sp.	

	Anoxybacillus kestanbolensis	
	Bacillus horneckiae	
	Brochothrix sp.	
	Paenibacillus sp.	
	Staphylococcus sp.	
	Staphylococcus haemolyticus	
	Staphylococcus pettenkoferi	
	Enterococcus sp.	
	Lactobacillus sp.	
	Turicibacter sp.	
	Clostridiales sp.	
	Lachnospiraceae sp.	
	Turicibacter sp.	
	Clostridiales sp.	
	Lachnospiraceae sp.	
	Eubacterium biforme	
	Arthrospira sp.	
	Caulobacter sp.	
	Rhizobiales sp.	
	Bradyrhizobiaceae sp.	
	Paracoccus aminovorans	
	Rhodospirillaceae sp.	
	Rickettsiales sp.	
	Carica papaya	
	Burkholderiaceae sp.	
	Variovorax paradoxus	
	Herbaspirillum sp.	
	Janthinobacterium lividum	
	Ralstonia sp.	
	Methylobacillus sp.	
	Rhodocyclaceae sp.	
	Rhodocyclaceae sp.	
	Aeromonadaceae sp.	
	Enterobacteriaceae sp.	
	Acinetobacter johnsonii	
	Enhydrobacter sp.	
	Pseudomonas sp.	
	Dyella sp.	
	Pseudoxanthomonas taiwanensis	
P4	Ellin6075 sp.	*Parabacteroides distasonis*
	Corynebacterium sp.	S24-7 sp.
	Friedmanniella sp.	*Elizabethkingia meningoseptica*
	Enterococcus sp.	*Pedobacter* sp.
	Coprococcus sp.	*Staphylococcus aureus*
	Rhodospirillaceae sp.	*Lactobacillus* sp.
		Turicibacter sp.
		Clostridiales sp.
		Allobaculum sp.
		Mycoplana sp.
		Rhizobiales sp.

		Bradyrhizobium sp.
		Hyphomicrobium sp.
		Sphingomonadaceae sp.
		Sutterella sp.
		Akkermansia muciniphila
P5	Actinomyces sp.	*Parabacteroides distasonis*
	Paenibacillus sp.	*Staphylococcus pettenkoferi*
	Helcococcus sp.	*Sphingomonas* sp.
	Peptoniphilus sp.	*Curvibacter* sp.
	Psychrobacter sanguinis	*Roseateles depolymerans*
	Akkermansia muciniphila	*Herbaspirillum* sp.
		Enterobacteriaceae sp.
P6	Streptophyta sp.	*Corynebacterium* sp.
	Planococcaceae sp.	*Prevotella* sp.
	Staphylococcus aureus	Bradyrhizobiaceae sp.
	Staphylococcus epidermidis	*Hyphomicrobium* sp.
	Streptococcus sp.	*Mesorhizobium* sp.
	Sphingomonadaceae sp.	*Sphingomonas* sp.
	Campylobacter ureolyticus	Comamonadaceae sp.
		Herbaspirillum sp.
		Aeromonadaceae sp.
P7	Rhizobiales sp.	*Corynebacterium* sp.
	Mesorhizobium sp.	*Bacteroides acidifaciens*
	Agrobacterium sp.	*Bacillus* sp.
		Staphylococcus epidermidis
		Bradyrhizobiaceae *sp.*
		Flexispira sp.
P8	*Corynebacterium simulans*	
	Bacillus sp.	
	Paenibacillus sp.	
	Planococcaceae sp.	
	Staphylococcus haemolyticus	
	Ruminococcus gnavus	
	Anaerococcus sp.	
	Finegoldia sp.	
	Bradyrhizobiaceae sp.	
	Sphingomonadaceae sp.	
	Comamonadaceae sp.	
	Haemophilus parainfluenzae	
P9	*Staphylococcus aureus*	*Bacteroides* sp.
	Clostridium sp.	*Bacteroides fragilis*
	Finegoldia sp.	*Prevotella* sp.
	Peptoniphilus sp.	*Butyricimonas* sp.
	Campylobacter ureolyticus	*Enterococcus* sp.
	Enterobacteriaceae sp.	*Vagococcus* sp.
	Enterobacter ludwigii	*Moryella* sp.

	Pseudomonas sp.	1-68 sp.<br>Bradyrhizobiaceae sp.<br>Alcaligenaceae sp.
P10	Planococcaceae sp.<br>*Clostridium* sp.<br>*Finegoldia* sp.<br>*Hyphomicrobium* sp.<br>*Rhodospirillaceae sp.*	*Bacteroides* sp.<br>*Prevotella* sp.<br>*Paenibacillus* sp.<br>*Staphylococcus* sp.<br>*Caloramator* sp.<br>*Bradyrhizobium* sp.<br>Phyllobacteriaceae sp.<br>*Agrobacterium* sp.<br>*Rhizobium* sp.<br>*Paracoccus aminovorans*<br>*Rhodobacter* sp.<br>Rickettsiales sp.<br>Sphingomonadales sp.<br>*Novosphingobium* sp.<br>Spirobacillales sp.<br>Enterobacteriaceae sp.
P13	Planococcaceae sp.<br>Gemellales sp.<br>Rhizobiales sp.<br>Oxalobacteraceae sp.	*Arcanobacterium* sp.<br>*Kocuria* sp.<br>*Bacteroides fragilis*<br>*Porphyromonas* sp.<br>*Prevotella* sp.<br>*Bacillus* sp.<br>*Moryella* sp.<br>*Finegoldia* sp.<br>Ellin329 sp.<br>Alcaligenaceae sp.<br>Xanthomonadaceae sp.<br>*Dyella* sp.
P14	*Mycobacterium llatzerense*<br>*Bacteroides uniformis*	Actinomyces sp.<br>*Nocardiopsis exhalans*<br>*Propionibacterium* sp.<br>*Bacteroides* sp.<br>*Bacteroides fragilis*<br>*Porphyromonas* sp.<br>*Butyricimonas* sp.<br>*Bacillus* sp.<br>*Paenibacillus* sp.<br>*Planococcus maitriensis*<br>*Staphylococcus aureus*<br>*Moryella* sp.<br>*Anaerococcus* sp.<br>*Finegoldia* sp.<br>*Peptoniphilus* sp.<br>*Gemmatimonas* sp.<br>*Arthrospira* sp.

		Caulobacter sp.<br>*Mycoplana* sp.<br>*Phenylobacterium* sp.<br>Ellin329 sp.<br>*Balneimonas* sp.<br>*Bradyrhizobium* sp.<br>Methylobacteriaceae sp.<br>*Methylobacterium* sp.<br>Rhizobiaceae sp.<br>*Paracoccus aminovorans*<br>Sphingomonadaceae sp.<br>*Novosphingobium* sp.<br>*Sphingomonas asaccharolytica*<br>Burkholderiales sp.<br>Alcaligenaceae sp.<br>*Sutterella* sp.<br>*Roseateles depolymerans*<br>Oxalobacteraceae sp.<br>*Herbaspirillum* sp.<br>Rhodocyclaceae sp.<br>Spirobacillales sp.<br>Shewanellaceae sp.<br>*Pseudomonas* sp.<br>Xanthomonadaceae sp.
P15	*Actinobaculum* sp.<br>*Corynebacterium* sp.<br>*Dermabacter* sp.<br>Streptophyta sp.<br>*Clostridium* sp.<br>*Anaerococcus* sp.<br>*Herbaspirillum* sp.<br>*Pseudomonas* sp.	Planococcaceae sp.<br>Gemellales sp.<br>Bradyrhizobiaceae sp.<br>*Sphingomonas* sp.
