# Supplementary material for: Measuring the microbiome of chronic wounds with use of a topical antimicrobial dressing – A feasibility study
Source: PLoS One. 2017 Nov 20;12(11):e0187728. doi: 10.1371/journal.pone.0187728 (PMC5695787; doi:10.1371/journal.pone.0187728)
Supplement: S2 File — (PDF) [file pone.0187728.s002.pdf]

Date: Tuesday, January 26, 2016 11:19:37 AM

Print Close

VIEW000072

1.1 Study Identification

*All questions marked by a red asterisk * are required fields. However, because the mandatory fields have been kept to a minimum, answering only the required fields may not be sufficient for the REB to review your application.*

Please answer all relevant questions that will reasonably help to describe your study or proposed research.

1.0 * **Short Study Title** *(restricted to 250 characters)*:
Molecular microbial ecology as a diagnostic tool to identify mode of action and new targets for oxidized silver wound dressings

2.0 * **Complete Study Title** *(can be exactly the same as short title)*:

Molecular microbial ecology as a diagnostic tool to identify mode of action and new targets for oxidized silver wound dressings

3.0 * **Select the appropriate Research Ethics Board** (*Detailed descriptions are available by clicking the HELP link in the upper right hand corner of your screen*):
HREB Biomedical

4.01 * **Is the proposed research:**
Funded (Grant, subgrant, contract, internal funds, donation or some other source of funding)

5.0 * **Name of Principal Investigator** *(at the University of Alberta, Covenant Health, or Alberta Health Services)*:
Benjamin Willing

6.0 **Investigator's Supervisor** *(required for applications from undergraduate students, graduate students, post-doctoral fellows and medical residents to Boards 1, 2, 3. HREB does not accept applications from student PIs)*

7.0 * **Type of research/study:**
Faculty/Staff Research

8.01 **Study Coordinators or Research Assistants:** People listed here can edit this application and will receive all HERO notifications for the study

Name	Employer
There are no items to display	

9.01 **Co-Investigators:** People listed here can edit this application but do not receive HERO notifications unless they are added to the study email list:

Name	Employer	Employer ID
There are no items to display		

10.01 **Study Team** *(Co-investigators, supervising team, other study team members)*: People listed here cannot edit this application and do not receive HERO notifications:

Last Name	First Name	Organization	Role/Area of Responsibility	Phone	Email
Kalan	Lindsay	Exciton Technologies Inc	Study coordination	780 248-5883	lkalan@excitontech.com
Suitor	Michele	Alberta Health Services	Nurse, patient engagement, sample collection	780-968-3738	michele.suitor@albertahealthservices.ca
Zhou	Mi	University of Alberta	Sample processing and analysis		mzhou2@ualberta.ca

1.2 Additional Approval

1.0 * **Departmental Review:**
AH Ag Food & Nutritional Sci

2.0 **Internal Review:**

1.3 Study Funding Information

1.0 * **Type of Funding:**

Grant (external)

If OTHER, provide details:

2.0 * **Indicate which office administers your award.** *(It is the PI's responsibility to provide ethics approval notification to any office other than the ones listed below)*
University of Alberta - Research Services Office (RSO)

If OTHER, provide details:

3.0 * Funding Source

3.1 Select all sources of funding from the list below:
NSERC - Natural Sciences And Engineering Research Council NSERC

3.2 If not available in the list above, write the Sponsor/Agency name(s) in full (you may add multiple funding sources):
There are no items to display

4.0 * Indicate if this research sponsored or monitored by any of the following:

Not applicable

If applicable, indicate whether or not the FDA Investigational New Drug number or FDA Investigational Device Exception is required:

The researcher is responsible for ensuring that the study complies with the applicable US regulations. The REB must also meet particular review criteria and this application will likely receive full board review, regardless of level risk.

1.4 RSO Managed Funding

1.0 If your funds are managed by Research Services Office (RSO), select the Project ID and title from the list below to facilitate release of your study funds. *(Not available yet)*

2.0 * To connect your ethics application with your funding: provide all identifying information about the study funding – multiple rows allowed. For Project ID, enter a Funding ID provided by RSO/PeopleSoft Project ID *(for example, RES0005638, G018903401, C19900137, etc).* Enter the corresponding title for each Project ID.

	Project ID	Project Title	Speed Code	Other Information
View	RES0020104	Molecular microbial ecology as a diagnostic tool to identify mode of action and new targets for oxidized silver wound dressings		

1.5 Conflict of Interest

1.0 * Are any of the investigators or their immediate family receiving any personal remuneration (including investigator payments and recruitment incentives but excluding trainee remuneration or

graduate student stipends) from the funding of this study that is not accounted for in the study budget?

Yes ◉ No

If YES, explain:

2.0 * Do any of investigators or their immediate family have any proprietary interests in the product under study or the outcome of the research including patents, trademarks, copyrights, and licensing agreements?

◉ Yes No

3.0 * Is there any compensation for this study that is affected by the study outcome?

Yes ◉ No

4.0 * Do any of the investigators or their immediate family have equity interest in the sponsoring company? (This does not include Mutual Funds)

Yes ◉ No

5.0 * Do any of the investigators or their immediate family receive payments of other sorts, from this sponsor (i.e. grants, compensation in the form of equipment or supplies, retainers for ongoing consultation and honoraria)?

Yes ◉ No

6.0 * Are any of the investigators or their immediate family, members of the sponsor's Board of Directors, Scientific Advisory Panel or comparable body?

Yes ◉ No

7.0 * Do you have any other relationship, financial or non-financial, that, if not disclosed, could be construed as a conflict of interest?

Yes ◉ No

If YES, explain:

Dr. Kalan is an employee of Exciton Technologies. She does not have any claim to ownership (not a share holder) or inclusion on patents.

Important

If you answered YES to any of the questions above, you may be contacted by the REB for more information or asked to submit a Conflict of Interest Declaration.

1.6 Research Locations and Other Approval

1.0 * List the locations of the proposed research, including recruitment activities. Provide name of institution or organization, town, or province as applicable

Westview Health Centre, Stony Plain, AB

2.0 * Indicate if the study will use or access facilities, programmes, resources, staff, students, specimens, patients or their records, at any of the sites affiliated with the following *(select all that apply)*:

Alberta Health Services Institutions and Facilities

Alberta Health Services Institutions and Facilities OUTSIDE the GREATER EDMONTON

List all facilities or institutions as applicable:

3.0

Multi-Institution Review

* 3.1 Has this study already received approval from another REB?

○ Yes ● No

4.0

Does this study involve pandemic or similar emergency health research?

○ Yes ● No

If YES, are you the lead investigator for this pandemic study?

○ Yes ○ No

5.0 If this application is closely linked to research previously approved by one of the University of Alberta REBs or has already received ethics approval from an external ethics review board*(s)*, provide the HERO study number, REB name or other identifying information. Attach any external REB application and approval letter in Section 7.1.11 – Other Documents.

2.1 Study Objectives and Design

1.0 Date that you expect to start working with human participants:

05/11/2013

2.0 Date that you expect to finish working with human participants, in other words, you will no longer be in contact with the research participants, including data verification and reporting back to the group or community:

30/04/2014

3.0 * Provide a lay summary of your proposed research suitable for the general public *(restricted to 300 words)*. If the PI is not affiliated with the University of Alberta, Alberta Health Services or Covenant

Health, please include institutional affiliation.

Chronic non-healing wounds considerably impact quaity of life in affected patients are a substantial burden on the Canadian health care system. Microbes colonizing a chronic wound play an important role in impeding effective healing. Chronic wounds are colonized by polymicrobial communities and no single orgnaism can be seen as causal. Only a small fraction of wound bacteria are cultured by diagnostic tests and studies have shown little agreement between culture and molecular based approaches, therefore an effective diagnostic for wound microbes is required. It is known that the composition of the microbial community associated with a wound changes as it heals although the causal relationship is somewhat unclear. Although not very effective in treating chronic non-healing wounds, antibiotics are often administered, contributing to concerns of antibiotic resistance. The wound dressing produced by Exciton Technologies Inc. (ETI) effectively aids in the healing process in chronic wounds through unknown mechanisms. ETI's wound dressings contain a combination of silver salts with three different valence, +1, +2 and +3 that have antimicrobial activity and are effective in reducing biofilm formation in vitro. However, it is not known how these silver salts impact microbial ecology of the wound and the role this plays in wound healing. The objectives of this research are to develop a new diagnostic tool based on molecular characterization of wound sites so as to predict how to best treat wounds and to identify new microbes to be targeted by ETI's technology. This project will utilize molecular microbial ecology for the asessment and evaluation fo topical silver interventions, gaining insight into the management of chronic infection. Substantiating the microbiota-modifying effectiveness of silver wound dressing, increasing clinician and patient understanding and improving clinical outcomes.

4.0 * **Provide a description of your research proposal including study objectives, background, scope, methods, procedures, etc)** *(restricted to 1000 words)*. **Footnotes and references are not required and best not included here. Research methods questions in Section 5 will prompt additional questions and information.**

Chronic non-healing wounds considerably impact quality of life in affected patients and are a substantial burden on the Canadian health care system. Variations in microbial colonization on wound sites suggest that there are two types of colonization on a wound; healing-compatible or incompatible. After completing in vitro testing, ETI identified that their silver oxysalt technology (exSALT®) is bacteriocidal against lab strains of known wound pathogens. However, wounds are polymicrobial in composition, and no single organism has been associated with non-healing wounds. The complexity of these infections needs to be tackled by studying the numerous microbes and their ecology using molecular methods, as only a small fraction of wound bacteria are cultured by diagnostic tests and studies have shown little agreement between culture and molecular based approaches. To identify the microbes in the wound bed affected by exSALT the microbial community must be studied directly from wound samples, as culture technology cannot replicate this complex community. Through this project we hope develop a diagnostic method to identify which deleterious organisms are targeted by exSALT® as well as those that are not, providing opportunities for more targeted

therapies. Efforts will lead to the development of a novel, low-cost, solution to prevention and treatment of chronic wounds affected by a polymicrobial infection.

Silver, in its metallic form and singly oxidized state, has been exploited for applications in a wide variety of applications including wound care devices. ETI is unique in that they are the first to use not only Ag^{+1}, but higher oxidation states of silver: Ag^{+2} and Ag^{+3}. The effect of silver ions on bacteria in culture has been performed, however, there are no studies examining the effect of silver, in any oxidized state, on the microbial ecology of the wound. ETI has developed a novel line of patent protected silver oxysalt composite materials. These compounds deliver antimicrobial and wound healing outcomes equal or greater than market leading silver dressings while only using a fraction the amount of total silver per dressing and providing increased patient comfort. **This study will utilize molecular microbial technology based diagnostic to characterize changes to wound microbial populations when treated with exSALT®.** Possible study subjects will be identified from new patients at the Westview Health Centre with chronic wounds. Patients that enroll in the study will receive standard care as provided by the staff at Westview Health Centre. For chronic wound patients this involves sharp debridement on a weekly basis. This is frequently followed by a swab of the wound bed for culture based diagnostics. Currently, exsalt SD7 wound dressings are applied to some patients, whereas in this study all patients will receive exsalt SD7 wound dressings. Swabs from patients prior to exsalt SD7 wound dressing application will act as control samples from each subject. Swabs will be taken following initial debridement, one day after application, and then weekly following debridement for up to 4 weeks for molecular analysis of microbial communities. We will also collect debridement tissue, which is normally discarded as biological waste. Along with debridement and swab samples, patient history, and the healing outcome data will be collected. Healing outcome will be measured using the Pixelere software currently in use at the clinic. Bacterial community composition will be assessed by barcoded pyrosequencing of bacterial 16S rRNA genes amplified from DNA isolated from wound swab and debridement samples using the 454 FLX platform as previously performed by the Willing lab. Because 23% of chronic wounds contain significant fungal populations, the fungal microbial community will also be assessed using fungal specific 18S rRNA primers and barcoded pyrosequencing. Our lab has established the tools to characterize microbial community composition in low microbial abundance samples (biopsies) and the correlation of imbalanced communities with select phenotypes.

5.0 **Describe procedures, treatment, or activities that are above or in addition to standard practices in this study area** *(eg. extra medical or health-related procedures, curriculum enhancements, extra follow-up, etc)*:

There will be no additional treatments or activities above or in addition to standard practices in this study area. The only additional procedures will involve collecting the additional specimens for examination by the researcher. Specimens are regularly collected in the study area and the procedure is not outside common practice. There will be no additional or undue discomfort to the study patients.

6.0 **If the proposed research is above minimal risk and is not funded via a competitive peer review grant or industry-sponsored clinical trial, the REB will require evidence of scientific review. Provide information about the review process and its results if appropriate.**

7.0 **For clinical research only, describe any sub-studies associated with this application.**
None

3.1 Risk Assessment

1.0 * **Provide your assessment of the risks that may be associated with this research:**
Minimal Risk - research in which the probability and magnitude of possible harms implied by participation is no greater than those encountered by participants in those aspects of their everyday life that relate to the research (TCPS2)

2.0 * **Select all that might apply:**

Description of Potential Physical Risks and Discomforts

No Participants might feel physical fatigue, e.g. sleep deprivation

No Participants might feel physical stress, e.g. cardiovascular stress tests

No Participants might sustain injury, infection, and intervention side-effects or complications

No The physical risks will be greater than those encountered by the participants in everyday life

Potential Psychological, Emotional, Social and Other Risks and Discomforts

No Participants might feel psychologically or emotionally stressed, demeaned, embarrassed, worried, anxious, scared or distressed, e.g. description of painful or traumatic events

No Participants might feel psychological or mental fatigue, e.g intense concentration required

No Participants might experience cultural or social risk, e.g. loss of privacy or status or damage to reputation

No Participants might be exposed to economic or legal risk, for instance non-anonymized workplace surveys

No The risks will be greater than those encountered by the participants in everyday life

3.0 * **Provide details of the risks and discomforts associated with the research, for instance, health cognitive or emotional factors, socio-economic status or physiological or health conditions:**
Because there will be no additional treatments or activities above or in

addition to standard practices in this study we do expect adverse effects of the study.

4.0 * **Describe how you will manage and minimize risks and discomforts, as well as mitigate harm:**
We will not be increasing risks or discomfort.

5.0 * **If your study has the potential to identify individuals that are upset, distressed, or disturbed, or individuals warranting medical attention, describe the arrangements made to try to assist these individuals. Explain if no arrangements have been made:**
Individuals in the study are already receiving medical attention, therefore this is not a possibility.

3.2 Benefits Analysis

1.0 * **Describe any potential benefits of the proposed research to the participants. If there are no benefits, state this explicitly:**
Beneficial responses in wound healing have been observed in patients receiving silver wound dressings. Patients receiving ETI's wound dressings may see improved healing over standard treatment.

2.0 * **Describe the scientific and/or scholarly benefits of the proposed research:**
Chronic non-healing wounds are a major health care burden in Canada (estimated at $3.9 billion yearly) and severely impact quality of life with considerable morbidity, prolonged hospitalization, pain and restricted mobility. Unfortunately, in a significant number of patients chronic non-healing wounds lead to amputation. Although not very effective in treating chronic non-healing wounds, antibiotics are often administered, contributing to concerns of antibiotic resistance. Canadian health care is therefore in urgent need of innovative, cost-effective, wound management tools for economical, best-in-class patient care/clinical outcomes. This project will utilize molecular microbial ecology as a new diagnostic tool to examine the effect of topical silver intervention on microbial ecology, gaining insight into the management of chronic infection. Results will substantiate the microbiota-modifying effectiveness of silver wound dressings and increase clinician and patient understanding to improving clinical outcomes. It will also provide new insights into the relationship between polymicrobial communities and chronic wounds.

3.0 **Benefits/Risks Analysis: Describe the relationship of benefits to risk of participation in the research:**
There is a lot of potential benefit for wound care practices and no risks associated with the study

4.1 Participant Information

1.0 * **Who are you studying? Describe the population that will be included in this study.**

Patients with chronic wounds. The population included in the study will be selected from new patients admitted for wound care to the Westview Health Centre.

2.0 * **Describe the inclusion criteria for participants (e.g. age range, health status, gender, etc.). Justify the inclusion criteria (e.g. safety, uniformity, research methodology, statistical requirement, etc)**

Age range: >18 <70

Wound history: Patients with chronic wounds (defined as >6 weeks non-healing)

Patient not on systemic antibiotic within past 3 months.

3.0 **Describe and justify the exclusion criteria for participants:**

Antibiotics can impact the wound microbiota, therefore will be exlcuded.

In this study we are only interested in chronic wounds.

4.0

* **Will you be interacting with human subjects, will there be direct contact with human participants, for this study?**

(•) **Yes** ( ) No

Note: No means no direct contact with participants, chart reviews, secondary data, interaction, etc.

If NO, is this project a chart review or is a chart review part of this research project?

( ) Yes ( ) No

5.0

Participants

How many participants do you hope to recruit *(including controls, if applicable)*

50

Of these how many are controls, if applicable *(Possible answer: Half, Random, Unknown, or an estimate in numbers, etc)*

50

If this is a multi-site study, for instance a clinical trial, how many participants *(including controls, if applicable)* **are expected to be enrolled by all investigators at all sites in the entire study?**

50

6.0 **Justification for sample size:**

Microbial populations in wounds can be quite variable between patients, therefore to gain insight into the relationship between treatment and

microbial population a substantial patient cohort is necessary. There are relatively few wound microbiome studies to date, but with an n between 23 and 30 significant conclusions were reached. To account for patients dropping out of the study 50 participants will be recruited.

7.0 **Does the research specifically target aboriginal groups or communities?**

( ) Yes (•) No

4.3 Recruit Potential Participants

1.0

Recruitment

* **1.1 Describe how you will identify potential participants (please be specific as to how you will find potentially eligible participants i.e. will you be screening AHS paper or electronic records, will you be looking at e-clinician, will you be asking staff from a particular area to let you know when a patient fits criteria, will you be sitting in the emergency department waiting room, etc.)**

New patients with chronic wounds that come in to Westview Health Centre for care that fit inclusion criteria will be asked to enrol in the study.

1.2 Once you have identified a list of potentially eligible participants, indicate how the potential participants' names will be passed on to the researchers AND how will the potential participants be approached about the research.

The nurse practitioner that identifies potential participants will ask the eligible participants directly as she is aware of the inclusion criteria.

1.3 How will people obtain details about the research in order to make a decision about participating? Select all that apply:

Researchers will contact potential participants

1.4 If appropriate, provide the locations where recruitment will occur *(e.g schools, shopping malls, clinics, etc.)*

2.0

Pre-Existing Relationships

2.1 Will potential participants be recruited through pre-existing relationships with researchers *(e.g. Will an instructor recruit students from his classes, or a physician recruit patients from her practice? Other examples may be employees, acquaintances, own children or family members, etc)***?**

( ) Yes (•) No

2.2 If YES, identify the relationship between the researchers and participants that could compromise the freedom to decline *(e.g. professor-student)***. How will you ensure that there is no undue pressure on the potential participants to agree to the study?**

3.0 **Outline any other means by which participants could be identified, should additional participants be needed** *(e.g. response to advertising such as flyers, posters, ads in newspapers, websites, email, listservs; pre-existing records or existing registries; physician or community organization referrals; longitudinal study, etc)*

4.0 **Will your study involve any of the following** *(select all that apply)*?
None of the above

4.5 Informed Consent Determination

1.0 *** Describe who will provide informed consent for this study (select all that apply). Additional information on the informed consent process is available at: http://www.pre.ethics.gc.ca/eng/policy-politique/initiatives/tcps2-eptc2/chapter3-chapitre3/#toc03-intro**

All participants have capacity to give free and informed consent

Provide justification for requesting a Waiver of Consent (Minimal risk only, additional guidance available at: http://www.pre.ethics.gc.ca/eng/policy-politique/initiatives/tcps2-eptc2/chapter3-chapitre3/#toc03-1b

2.0 **How is participant consent to be indicated and documented? Select all that apply:**

Signed consent form

Except for "Signed consent form" use only, explain how the study information will be communicated and participant consent will be documented. Provide details for EACH of the option selected above:

3.0 **Authorized Representative, Third Party Consent, Assent**

3.1 Explain why participants lack capacity to give informed consent *(e.g. age, mental or physical condition, etc.)*

3.2 Will participants who lack capacity to give full informed consent be asked to give assent?
Yes No

Provide details. IF applicable, attach a copy of assent form(s) in the Documentation section.

3.3 In cases where participants (re)gain capacity to give informed consent during the study, how will they be asked to provide consent on their own behalf?

4.0 **What assistance will be provided to participants, or those consenting on their behalf, who have special needs?** *(E.g. non-English speakers, visually impaired, etc)*:

5.0 * **If at any time a participant wishes to withdraw, end, or modify their participation in the research or certain aspects of the research, describe how their participation would be ended or changed.**
If patients wish to withdraw from the study they can request for their samples to be destroyed. Written or verbal request of removal will be accepted from the nurse practitioner.

6.0 **Describe the circumstances and limitations of data withdrawal from the study, including the last point at which it can be done:**
The patient may withdraw from the study at any point before the final sample is collected at week 4.

7.0 **Will this study involve any group(s) where non-participants are present? For example, classroom research might involve groups which include participants and non-participants.**
○ Yes ◉ No

5.1 Research Methods and Procedures

Some research methods prompt specific ethic issues. The methods listed below have additional questions associated with them in this application. If your research does not involve any of the methods listed below, ensure that your proposed research is adequately described in Section 2.0: Study Objectives and Design or attach documents in Section 7.0 if necessary.

1.0 * **This study will involve the following** *(select all that apply)*
The list only includes categories that trigger additional page(s) for an online application. For any other methods or procedures, please indicate and describe in your research proposal in the Study Summary, or provide in an attachment:
Sound or Image Data (other than audio or video-recorded interviews)
Medical Devices
Health and Biological Specimen Collection or Use of Previously Collected Specimens
Biohazardous Substances

2.0 * **Is this study a Clinical trial? (Any investigation involving participants that evaluates the effects of one or more health-related interventions on health outcomes?**
○ Yes ◉ No

3.0 **If you are using any tests in this study diagnostically, indicate the member(s) of the study team who will administer the measures/instruments:**

Test Name	Test Administrator	Organization	Administrator's Qualification

Test Name Test Administrator Organization Administrator's Qualification
There are no items to display

4.0 **If any test results could be interpreted diagnostically, how will these be reported back to the participants?**

5.6 Sound or Image (other than audio- or video-recorded interviews) or Material Created by Participants

1.0 **Explain if consent obtained at the beginning of the study will be sufficient, or if it will be necessary to obtain consent at different times, for different stages of the study, or for different types of data:**
Consent at the beginning is sufficient as photographs will be taken as part of the normal care practice. Westview Health Centre currently administers their own consent forms for digital photography of wounds.

2.0 **At what stage, if any, can a participant withdraw his/her material?**
Any stage until final sample collection.

3.0 **If you or your participant's audio- or video-records, photographs, or other materials artistically represent participants or others, what steps will you take to protect the dignity of those that may be represented or identified?**
There will not be any identification made available and pictures will not be identifiable except by patient ID.

4.0 **Who will have access to this data? For example, in cases where you will be sharing sounds, images, or materials for verification or feedback, what steps will you take to protect the dignity of those who may be represented or identified?**
All team members will have access to this information. Outside of the nurse practitioner, the identity of the patients will be unknown.

5.0 **When publicly reporting data or disseminating results of your study (eg presentation, reports, articles, books, curriculum material, performances, etc) that include the sounds, images, or materials created by participants you have collected, what steps will you take to protect the dignity of those who may be represented or identified?**
All patient identifiers will be removed from all data reporting.

6.0 **What opportunities are provided to participants to choose to be identified as the author/creator of the materials created in situations where it makes sense to do so?**
N/A

7.0 **If necessary, what arrangements will you make to return original materials to participants?**
N/A

5.9 Investigational Drugs, Devices, Biologics, Vaccines or Natural Health Products

1.0 **List all the investigational drugs, biologics, vaccine, natural health products, or devices used in the study. Enter the Health Canada No Objection Letter (NOL) control number and date of approval if available for the initial application and subsequent NOLs for amendments. Upload the NOL letter in the Documentation Section of your application.**

	Name	Manufacturer	Type	Health Canada Approval Status	NOL Control Number	Date
View	exsalt SD7 wound dressing	Exciton Technologies Inc	Device	NOL Enclosed		

5.11 Health and Biological Specimen Collection

1.0 * **Indicate health or biological specimen(s) that will be collected** *(for example, body tissues or fluids, be specific)*:
wound debridement tissue and swabs

2.0 * **This study will involve the following** *(select all that apply)*:

Collection of sample for immediate use

If OTHER, provide details:

3.0 **Explain how the specimen will be collected:**
Debridement tissue will be collected following standard protocols by nurse practitioner. Swabs will be taken of the wound bed following debridement.

4.0 **Explain HOW the specimen will be stored:**
The specimens will be stored at -80 degrees C in a screw top tube.

Explain HOW LONG the specimens will be stored:
Maximum of 5 years.

5.0 **Explain WHERE the specimens will be stored** *(e.g. include information if the specimens will be sent out of the province)*:
In the laboratory of Dr. Willing.

6.0 **Specify all intended uses of collected specimen:**
DNA extraction and characterization of microbial communities.

5.13 Biohazard Safety

1.0 **ONLY FOR AMENDMENT OR RENEWAL**: If this application is for the amendment or renewal of a pre-existing clinical study: have new biohazards and/or manipulations been added to the research that were not identified in the original study protocol? For a new study, please select "Not Applicable."
*

Not Applicable

If you selected **NO**, this amendment or renewal is exempt from requiring further review by the EHS Biosafety Division and the original biohazard approval remains valid. You do not need to respond to any of the questions below.

If you selected **YES**, this amendment or renewal is considered new research - please respond to question 2.0 below.

2.0

Will your research involve the use of one or more of the following? Provide a response for each item.

Answer	Description
Yes (•) **No**	Risk group 2, 3 or 4 viruses, bacteria, fungi, parasites or eukaryotic cell lines
(•) **Yes** No	Environmental specimens suspected to contain risk group 2, 3 or 4 microbes
Yes (•) **No**	Large-scale single volume culture in excess of 10 litres for any microbe or eukaryotic cell line
Yes (•) **No**	Microbial toxins
(•) **Yes** No	Human clinical specimens, including blood or other body fluids, or primary culture of human cells
Yes (•) **No**	Xenotransplant studies involving vertebrate donors and/or recipients
Yes (•) **No**	Genetic therapy studies involving vertebrate donors and/or recipients
Yes (•) **No**	Genetic manipulation involving virulence genes from risk group 2, 3 or 4 microbes, mammalian oncogenes, mammalian cytokine or interleukin genes, or microcide resistance genes
Yes (•) **No**	Genetic manipulations involving the use of recombinant vector systems based on lentivirus, adenovirus, retrovirus or herpesvirus backbones

If you answered YES to any of the above, the online system will forward your project information to the Biosafety Division for review and you will get notified regarding the issue of an auxiliary Biohazards approval.

6.1 Data Collection

1.0 * **Will the researcher or study team be able to identify any of the participants at any stage of the study?**
(•) **Yes** No

2.0

Will participants be recruited or their data be collected from Alberta Health Services or Covenant Health or data custodian as defined in the Alberta Health Information Act?
(•) **Yes** No

Important: Research involving health information must be reviewed by the Health Research Ethics Board.

3.0 **Primary/raw data collected will be** *(check all that apply)*:
All personal identifying information removed (anonymized)

4.0 **If this study involves secondary use of data, list all original sources:**

5.0 **In research where total anonymity and confidentiality is sought but cannot be guaranteed** *(eg. where participants talk in a group)* **how will confidentiality be achieved?**

6.2 Data Identifiers

1.0 * **Personal Identifiers:** will you be collecting - at any time during the study, including recruitment - any of the following *(check all that apply)*:

Full Date of Birth
Year of Birth
Age at time of data collection
Other

If OTHER, please describe:
gender

2.0 **Will you be collecting - at any time of the study, including recruitment of participants - any of the following** *(check all that apply)*:

Other

If OTHER, please describe:
Data from medical record (patient history)

3.0 * **If you are collecting any of the above, provide a comprehensive rationale to explain why it is necessary to collect this information:**
We will need to collect wound history as it will impact on the microbial composition as well as other factors that will impact wound healing such as diabetes and blood pressure.

4.0 **If identifying information will be removed at some point, when and how will this be done?**
In the data collection software at Westview Health Centre. Prior to information access by Drs. Willing, Kalan and Zhou.

5.0 * **Specify what identifiable information will be RETAINED once data collection is complete, and explain why retention is necessary. Include the retention of master lists that link participant identifiers with de-identified data:**
No identifiable information will be retained.

6.0 **If applicable, describe your plans to link the data in this study with data associated with other studies (e.g within a data repository) or with data belongong to another organization:**
None

6.3 Data Confidentiality and Privacy

1.0 * **How will confidentiality of the data be maintained? Describe how the identity of participants will be protected both during and after research.**
Researchers will never be given access to patient identity.

2.0 **How will the principal investigator ensure that all study personnel are aware of their responsibilities concerning participants' privacy and the confidentiality of their information?**
Only one member of the study team will have access to this information and they are already aware of patient-care confidentiality requirements.

3.0

External Data Access

* **3.1 Will identifiable data be transferred or made available to persons or agencies outside the research team?**
○ Yes ◉ No

3.2 If YES, describe in detail what identifiable information will be released, to whom, why they need access, and under what conditions? What safeguards will be used to protect the identity of subjects and the privacy of their data.

3.3 Provide details if identifiable data will be leaving the institution, province, or country *(eg. member of research team is located in another institution or country, etc.)*

6.4 Data Storage, Retention, and Disposal

1.0 * **Describe how research data will be stored, e.g. digital files, hard copies, audio recordings, other. Specify the physical location and how it will be secured to protect confidentiality and privacy.** *(For example, study documents must be kept in a locked filing cabinet and computer files are encrypted, etc. Write N/A if not applicable to your research)*
All patient information that is associated with patient identification will be protected by encryption of computer files by Westview Health Centre.

2.0 * **University policy requires that you keep your data for a minimum of 5 years following completion of the study but there is no limit on**

data retention. Specify any plans for future use of the data. If the data will become part of a data repository or if this study involves the creation of a research database or registry for future research use, please provide details. *(Write N/A if not applicable to your research)*
We do not plan to destroy the data, but do not have any current plans to include it in a registry or otherwise.

3.0 **If you plan to destroy your data, describe when and how this will be done? Indicate your plans for the destruction of the identifiers at the earliest opportunity consistent with the conduct of the research and/or clinical needs:**

7.1 Documentation

Add documents in this section according to the headers. Use Item 11.0 "Other Documents" for any material not specifically mentioned below.

Sample templates are available in the REMO Home Page in the **Forms and Templates**, or by clicking HERE.

1.0 **Recruitment Materials:**

Document Name	Version	Date	Description
There are no items to display			

2.0 **Letter of Initial Contact:**

Document Name	Version	Date	Description
There are no items to display			

3.0 **Informed Consent / Information Document(s):**

3.1 What is the reading level of the Informed Consent Form(s):
automated readability index of 5.5

3.2 Informed Consent Form(s)/Information Document(s):

Document Name	Version	Date	Description
participant consent form_Nov 4 13.pdf | History	0.08	14/11/2013 09:14	

4.0 **Assent Forms:**

Document Name	Version	Date	Description
There are no items to display			

5.0 **Questionnaires, Cover Letters, Surveys, Tests, Interview Scripts, etc.:**

Document Name	Version	Date	Description

Document Name	Version	Date	Description
There are no items to display			

6.0 **Protocol:**

Document Name	Version	Date	Description
protocol | History	0.01	04/10/2013 13:45	

7.0 **Investigator Brochures/Product Monographs** *(Clinical Applications only)*:

Document Name	Version	Date	Description
Product info | History	0.01	04/10/2013 13:47	

8.0 **Health Canada No Objection Letter** *(NOL)*:

Document Name	Version	Date	Description
There are no items to display			

9.0 **Confidentiality Agreement:**

Document Name	Version	Date	Description
There are no items to display			

10.0 **Conflict of Interest:**

Document Name	Version	Date	Description
There are no items to display			

11.0 **Other Documents:**

For example, Study Budget, Course Outline, or other documents not mentioned above

Document Name	Version	Date	Description
There are no items to display			

Final Page

You have completed your ethics application! Please select "Exit" to go to your study workspace.

This action will NOT SUBMIT the application for review.

Only the Study Investigator can submit an application to the REB by selecting the "SUBMIT STUDY" button in My Activities for this Study ID: Pro00042930.

You may track the ongoing status of this application via the study workspace.

Please contact the REB Coordinator with any questions or concerns.
